# The Resistance of Oilseed Rape Microspore-Derived Embryos to Osmotic Stress Is Associated With the Accumulation of Energy Metabolism Proteins, Redox Homeostasis, Higher Abscisic Acid, and Cytokinin Contents

**DOI:** 10.3389/fpls.2021.628167

**Published:** 2021-06-11

**Authors:** Milan O. Urban, Sébastien Planchon, Irena Hoštičková, Radomira Vanková, Peter Dobrev, Jenny Renaut, Miroslav Klíma, Pavel Vítámvás

**Affiliations:** ^1^Crop Research Institute, Plant Stress Biology and Biotechnology, Prague, Czechia; ^2^Luxembourg Institute of Science and Technology, “Environmental Research and Innovation,” (ERIN) Department, Belvaux, Luxembourg; ^3^Department of Plant Production and Agroecology, University of South Bohemia in Ceské Budějovice, Ceské Budějovice, Czechia; ^4^Laboratory of Hormonal Regulations in Plants, Institute of Experimental Botany of the Czech Academy of Sciences, Prague, Czechia

**Keywords:** microspore, 2D-DIGE, osmotic stress, *Brassica napus*, screening, RT-qPCR

## Abstract

The present study aims to investigate the response of rapeseed microspore-derived embryos (MDE) to osmotic stress at the proteome level. The PEG-induced osmotic stress was studied in the cotyledonary stage of MDE of two genotypes: Cadeli (D) and Viking (V), previously reported to exhibit contrasting leaf proteome responses under drought. Two-dimensional difference gel electrophoresis (2D-DIGE) revealed 156 representative protein spots that have been selected for MALDI-TOF/TOF analysis. Sixty-three proteins have been successfully identified and divided into eight functional groups. Data are available *via* ProteomeXchange with identifier PXD024552. Eight selected protein accumulation trends were compared with real-time quantitative PCR (RT-qPCR). Biomass accumulation in treated D was significantly higher (3-fold) than in V, which indicates D is resistant to osmotic stress. Cultivar D displayed resistance strategy by the accumulation of proteins in energy metabolism, redox homeostasis, protein destination, and signaling functional groups, high ABA, and active cytokinins (CKs) contents. In contrast, the V protein profile displayed high requirements of energy and nutrients with a significant number of stress-related proteins and cell structure changes accompanied by quick downregulation of active CKs, as well as salicylic and jasmonic acids. Genes that were suitable for gene-targeting showed significantly higher expression in treated samples and were identified as phospholipase D alpha, peroxiredoxin antioxidant, and lactoylglutathione lyase. The MDE proteome profile has been compared with the leaf proteome evaluated in our previous study. Different mechanisms to cope with osmotic stress were revealed between the genotypes studied. This proteomic study is the first step to validate MDE as a suitable model for follow-up research on the characterization of new crossings and can be used for preselection of resistant genotypes.

## Introduction

Plant breeding is focused on the permanent increase of crop production to meet the needs of an ever-growing world population, improving food quality to ensure long and healthy life, and to address the problems of global warming and environmental pollution, together with the challenges associated with developing novel biofuel sources. A combination of different approaches (mechanistic physiological understanding, -omics, association mapping, envirotyping, gene editing, and other tools) need to be utilized to improve significantly the abiotic stress resistance of crops in the field (Mittler and Blumwald, 2010). It is worthy to study contrasting genotypes and search for proteins potentially correlated with abiotic stress adaptability and acclimation in order to characterize the responsiveness of individual genotypes to multiple environmental parameters (Frenck et al., 2011; Claeys et al., 2014).

*Brassica napus* (winter oilseed rape), the second most-grown oil crop in the world, is the major oilseed crop in temperate regions of Europe and China (Hu et al., 2011). Several proteomics articles and reviews on stress-treated plants from the *Brassicaceae* family have been published (Subramanian et al., 2005; Devouge et al., 2007; Zhu et al., 2010, 2012; Bandehagh et al., 2011; Demartini et al., 2011; Fernandez-Garcia et al., 2011; Gan et al., 2013; Chu et al., 2015; Shen et al., 2015), including integrated proteomic/transcriptomic approach (Kubala et al., 2015; Shokri-Gharelo and Noparvar, 2018; Zhang et al., 2019; Schiessl et al., 2020). However, despite the importance of oilseed rape worldwide, between the years 2005 and 2021, only four drought-focused comparative proteomic studies were aimed at *B. napus* [seedling roots (Mohammadi et al., 2012), 20-day-old seedlings (Koh et al., 2015), and mature plants (Wang et al., 2016; Urban et al., 2017)]. NaCl and PEG solution proteomic analysis on 15-day-old seedlings of one cultivar was studied by Luo et al. (2015).

Microspore embryogenesis (the formation of embryos from immature pollen grains) is a unique system that speeds up breeding programs and, in the context of developmental biology, provides an excellent tool for embryogenesis to be investigated in greater detail. For some species, isolated microspore culture protocols have been well-established and are routinely used in laboratories for developing new varieties as well as for basic research areas, such as genomics, gene expression, and genetic mapping (Ferrie and Caswell, 2011). The last few years have provided ample evidence that has allowed researchers of Brassica to markedly increase their understanding of the molecular and subcellular changes underlying the early events of totipotency that permit microspores to develop into embryos and facilitate their transformation (Ahmadi et al., 2018). Isolated plant microspores can be diverted from their normal gametophytic pathway toward sporophytic development and used also in stress-responsive studies. In this shift transformation, the osmolality of growth media plays a significant role (Yadollahi et al., 2011), besides other stresses such as heat or cold (Prem et al., 2012) shock, starvation (Najafabadi et al., 2015), or endogenous auxin biosynthesis (Rodriguez-Sanz et al., 2015).

More than 340 and 78 article topics and titles, respectively, focused on “*B. napus* + microspore,” were published in the 2005–2020 period (Web of Science). Recently, Kitashiba et al. (2016) have identified loci associated with embryo yield in microspore culture of *Brassica rapa*. Daurova et al. changed microspore-derived embryos (MDE) fatty acid composition (Daurova et al., 2020). Genes associated with the induction and the establishment of embryogenesis in isolated microspores were studied by Malik et al. (2007), and Tsuwamoto et al. (2007). The potential involvement of specific ROP GTPases in early-stage microspore culture responses was studied by Chan and Pauls (2007). By comparing intervarietal substitution lines, eight genomic regions containing genetic factors controlling the rate of a direct embryo to plant conversion in rapeseed were identified by Kampouridis et al. (2016). *Via* label-free proteomics, Su et al. studied proteomic variations on cabbage embryo induction after short-term heat shock treatment (Su et al., 2020). Whittle et al. presented comparative transcriptional profiles of ovule, microspores, and pollen in *B. napus* (Whittle et al., 2010). Microspore embryogenesis constitutes a convenient system for studying the mechanisms underlying cell reprogramming and embryo formation. However, a comparative proteomic study aimed at already developed MD embryos and, besides, individual microspores in *B. napus* is missing.

In *B. napus*, doubled haploid (DH) production was first described in 1975 by Thomas and Wenzel (1975). DH production is a valuable technology in modern plant breeding of oilseed rape (Cegielska-Taras et al., 2008; Kaczmarek et al., 2008; Germana, 2011). Only a small part of Brassica MDEs will regenerate to the functional DH plants; therefore, the possibility of early, concise, and relevant detection of resistant genotypes can increase the effectivity of the whole process. Rapeseed DHs, which are haploids that have undergone chromosome duplication, represent an attractive biotechnological method to accelerate plant breeding by the production of entirely homozygous lines.

The novelty of this study is that the osmotic stress responses of two contrasting genotypes (their MDEs) are compared on different levels. The main aim of this study is to screen for proteins in oilseed rape MDE that are differentially regulated under osmotic stress, to characterize embryo hormonal profiles, and to explore in detail the mechanism of this response to low water potential in the growing medium during the MDE development. To date, this is the first proteomic study on PEG-influenced MDE. Different mechanisms to cope with osmotic stress were revealed between the genotypes studied. The wish of authors is to revalidate the MDE system as a suitable screening tool for early/mass selection of resistant DH embryos (e.g., using real-time quantitative PCR, RT-qPCR, from one cotyledon while the rest is cultivated later on). The comparison of MDE proteomic results with our previous leaf proteomic study (Urban et al., 2017) is also presented.

## Materials and Methods

### Samplings and Measurements

Seeds of two winter oilseed rape (*B. napus*. L.) cultivars (cvs.), Cadeli (D), and Viking (V) were germinated, and plants were cultivated under controlled conditions according to protocol described by Urban et al. (2017). After vernalization, plants were cultivated until the flower-bud stage as described below. For detailed information about cultivars, refer Supplementary Table 1.

### Microspore Culture Treatment With Antimitotic Agents

Microspore cultures were prepared according to the basic protocol of Klima et al. (2008). In short: young flower buds with microspores at mid-uninucleate and late-uninucleate developmental stages were collected from donor plants grown under controlled conditions in a culture chamber (light intensity, 84 μmol m^−2^ s^−1^; at 22/20°C, day/night; and photoperiod, 16/8 h). Microspores were isolated from flower buds after the microspore developmental stage observation. Freshly isolated and purified microspores were resuspended in the NLN liquid medium (Klima et al., 2008), supplemented with corresponding amounts of particular doubling agent stock solutions to get the final concentration of trifluralin 10 μmol/dm^3^. Microspores in 60 mm plastic Petri dishes containing 6 cm^3^ of suspension were incubated for 18 h at 30°C in the dark. After incubation, the microspores were purified by centrifugation, resuspended in a fresh NLN medium, and cultivated in the dark at 30°C with an antimitotic agent. After 3 weeks, embryos at the torpedo and early cotyledonary stage (at least 2 mm in length) were on the Petri dishes placed on a shaker (70 rpm) under continuous light at 22°C until embryos started growing and turned green. To lower the medium water potential (for treated medium), sterile aqueous PEG 4000 30% w/v (Sigma-Aldrich, 95904) solution was poured on the top of the solid differentiation medium (DM; Klima et al., 2008) with benzyl amino purine (0.2 mg/dm^3^), indole-3-acetic acid (0.2 mg/dm^3^), and 2% sucrose, solidified by 0.8% agar and then poured out after 24 h, according to protocol described by Verslues et al. (2006). Only traces of PEG are present in the final DM. That way prepared PEG is inert, non-ionic solutes able to decrease the osmotic pressure of tissues or media. The media low-water potential is stable for over a month (data not shown). The control DM was not treated. The pH of DM and PEG solution was adjusted to pH 5.8 just after autoclaving. Cotyledonary embryos of at least 4 mm in length were then transferred to a solid PEG-free treated (osmotically activated plates) or non-treated DM and maintained at 22/20°C, with photoperiod 10/14 h, and a light intensity of 300 μmolm^−2^s^−1^. After 1 day (for transcriptomic and hormonal study, C1 for control and S1 for a treated variant, respectively), and 7 days (for transcriptomic, hormonal, and proteomic studies, C2 for control and S2 for a treated variant, respectively), of cotyledonary embryo cultivation at control DM or PEG-treated DM, one-third of the bulked embryos was cleaned from media, weighed, and frozen immediately in liquid nitrogen for further protein extraction; then, two-third was cleaned, weighed, and frozen in liquid nitrogen for transcriptomic and hormonal study; and the last part was used for biomass accumulation. Only embryos similar in shape, color, and size were carefully selected and used for the study. Three independent biological replicates (MDE cultures established individually in time) with five technical repetitions (Petri dishes) were performed for each treatment. To prevent any possible osmotically-driven differences between dishes, we used half of each dish for both cultivars. MDE weight gain was calculated as a fresh weight difference from each repetition between the first and the last days of the treatment after MDE was cleaned from the medium. From each repetition (Petri dish), ~2 g of fresh MDE was used for further analyses. The repetitions were bulked together (after previous shape/size/stage verification) as one biological replicate. Each replicate (in total 10 g from five repetitions, fresh weight) was then immediately divided into three parts for further analyses.

### Protein Extraction and Two-Dimensional Difference Gel Electrophoresis (2D-DIGE) Analysis

Total soluble proteins were extracted from embryos as described in Wang et al. (2003), with some modifications as described in detail in Urban et al. (2017). Dry protein pellets were resolved in a lysis buffer according to GE Healthcare manual for 2D-DIGE analysis, pH of the solution was adjusted to 8.5 by 50 mM NaOH, and protein concentration was determined by 2D Quant kit (GE Healthcare; Sigma-Aldrich GE80-6483-56). The protein samples (15 μg) were labeled with CyDye® minimal dyes (GE Healthcare; Sigma-Aldrich GE25-8008-61) according to the instructions of the manufacturer. Samples were run on 11 cm IPG strips with a pI range of 5–8. Image capture of 1 mm thick gels was done using the PharosFX Plus (Bio-Rad) at a resolution of 600 dpi. Densitometric analysis of scanned images was carried out using PDQuest Advanced 8.0.1 (Bio-Rad). Protein spot normalization was carried out using the local regression model, and spot manual editing was carried out using a group consensus tool. The differentially abundant protein spots (characterized as those with at least a 2-fold change; *p* < 0.05 determined by Student's *T*-test) were chosen for spot excision (ExQuest Spot Cutter; Bio-Rad) and identification from preparative gels (2-DE of 200 μg of an internal standard sample) stained by Bio-Safe Coomassie G-250 stain (Bio-Rad; Sigma-Aldrich B0770). Each of three biological replicates of protein samples was created as bulk from five technical repetitions. Samples of control Viking (VC) and treated Cadeli (DS) were dyed four times and samples of control Cadeli (DC) and treated Viking (VS) were dyed six times. Cy3- and Cy5-labeled samples were randomly combined, and the Cy2-labeled internal standard was added to form a mixed sample for loading onto an IPG strip.

### The MS-Based Spot Identification and Database Search

For protein identification, the excised proteins were processed as described in Guerra-Guimaraes et al. (2015). Briefly, each sample was washed initially in 50 mM ammonium bicarbonate solution, containing 50% (v/v) methanol and dehydrated using 75% (v/v) acetonitrile (ACN) solution. Proteins were then digested in 8 μL of trypsin Gold (Promega; 5 ng/μL trypsin in 20 mM ammonium bicarbonate). After extraction with 50% (v/v) ACN, containing. 0.1% (v/v) trifluoroacetic acid (TFA), the peptides were dried at 50°C and spotted on MALDI-TOF target plates. A volume of 0.7 mm^3^ of 7 mg/cm^3^ α-cyano-4-hydroxycinnamic acid in 50% (v/v) ACN, containing 0.1% (v/v) TFA was added. A MALDI peptide mass spectrum was acquired using the AB Sciex 5800 TOF/TOF (AB Sciex, Foster City, CA, USA), and the 10 most abundant peaks, excluding known contaminants, were selected and fragmented. The ProteinPilot™ software 4.0.8085 was used for database searches with an in-house MASCOT platform (version 2.3, Matrix Science, www.matrixscience.com, London, UK). All proteins were identified by a search against NCBInr database 20151110 (76068736 sequences; 27,658,295,194 residues) with the taxonomy Viridiplantae (http://www.ncbi.nlm.nih.gov) containing 3,269,297 sequences and downloaded on November 15, 2016. All searches (combined MS and 10 MS/MS spectra) were carried out using a mass window of 100 ppm for the precursor and 0.5 Da for the fragments. During the different searches, the following parameters were defined: maximum two missed cleavages, fixed carbamidomethylation of cysteine, variable oxidation of methionine or tryptophan, and tryptophan to kynurenine or double oxidation to N-formylkynurenine, an unrestricted protein mass, and mass value set to monoisotopic. The 980 spots were found on each gel on average. Out of all differentially abundant protein spots (894 normalized spots, Boolean union of all normalized spots, revealing at least a 2-fold change at a 0.05 level determined by Student's *T*-test), just 212 spots were assessed by PDQuest with quantitative change of at least ± 2-fold. From these, those present in at least 80% of gels and revealing <50% variability in their SD density values relative to the mean sample values have been selected for protein spot excision (156 spots). All identification was manually validated, and extra precursors were selected for fragmentation if the obtained data were judged as insufficient. When high-quality spectra were not matched to sequences, a sequence was determined manually, and, in the current data set, it could be linked to the identified protein by allowing for more missed cleavages, semitryptic peptides, or specific modifications. At least three peptides with a score > 20, or at least two peptides with a score > 30, or at least one peptide with a score > 50 and 1 > 20, or, finally, a single peptide with a score > 90 were considered as significant (*p* < 0.05). Only the spots with a single (unique) and significant protein identification were considered for further bioinformatics analyses. The spots with multiple protein identification were not considered in the study and were all discarded.

### RNA Isolation and RT-qPCR

Samples for studying the expression of genes identified to encode osmotically induced proteins were collected as a bulk of three replicates (three biological replicates per five technical repetitions) and were stored at −80°C. Total RNA was extracted using the RNeasy plant mini kit (Qiagen) according to the instructions of the manufacturer. Contaminating DNA was removed using the DNA-free^TM^ DNA Removal Kit (Ambion; ThermoFisher Scientific AM1906). RNA was quantified using spectrophotometric measurements (OD260), and sufficient quality was assessed (OD260/280 ratio and OD260/230 ratio) by BioSpec Nano (Shimadzu). Total RNA was stored at −80°C. Complementary DNA templates were prepared using Standard Reverse Transcription Protocol (Promega) and stored at −20°C. The RT-qPCR was performed on the QuantStudio™ 6 Flex Real-Time PCR System (Applied Biosystems) using Power SYBR® Green PCR Master Mix (Applied Biosystems; ThermoFisher Scientific 4368708) in a 96-well reaction plate using parameters recommended by the manufacturer (2 min at 50°C, 10 min at 95°C, and 40 cycles of 15 s 95°C, 1 min of 60°C, 15 s at 95°C, 1 min at 60°C, and 15 s at 95°C). The three replicates and no-template controls were included. The specificity of amplification was determined by dissociation curve analyses. A comparative threshold cycle method was applied to determine relative concentrations of mRNA. The primers used are shown in SI 5 (Excel Supplementary Data). All the gene expression levels were normalized to Actin gene expression (BnAct), as a reference gene, and the obtained data were normalized to BnAct using the ΔΔCT method according to Livak and Schmittgen (2001). The reference gene was selected by using geNorm (Vandesompele et al., 2002). CT value of the Catalase gene (CAT4) in C1—cultivar D was used as a calibrator sample.

### Hormone Analysis

Embryo samples were purified and analyzed according to protocols described by Dobrev and Kaminek (2002) and Dobre and Vankova (2012). Samples were homogenized with a ball mill (MM301, Retsch) and extracted in pre-cold (−20°C) methanol/water/formic acid (15/4/1 v/v/v). The extracted samples were left at −20°C overnight. The following labeled internal standards (10 pmol/sample) were added: ^13^C_6_-IAA, ^2^H_2_-OxIAA (Cambridge Isotope Laboratories); ^2^H_4_-SA (Sigma-Aldrich); ^2^H_3_-PA (phaseic acid), ^2^H_3_-DPA (dihydrophaseic acid), ^2^H_4_-7OH-ABA, ^2^H_5_-ABA-GE (ABA-glucosyl ester) (NRC-PBI), ^2^H_6_-ABA, ^2^H_5_-JA, ^2^H_5_-transZ, ^2^H_5_-transZR, ^2^H_5_-transZ7G, ^2^H_5_-transZ9G, ^2^H_5_-transZOG, ^2^H_5_-transZROG, ^2^H_5_-transZRMP, ^2^H_3_-DHZ, ^2^H_3_-DHZR, ^2^H_3_-DZRMP, ^2^H_7_-DZOG, ^2^H_3_-DHZ9G, ^2^H_7_-DZOG, ^2^H_6_-iP, ^2^H_6_-iPR, ^2^H_6_-iP7G, ^2^H_6_- iP9G, and ^2^H_6_-iPRMP (Olchemim). The extract was centrifuged (17,000 *g*, 4°C, 20 min) to remove solid debris. It was then concentrated using an Alpha RVC vacuum centrifuge (Christ; 40°C, 15 mbar, 1.5 h). Extracts were purified using a mixed-mode reverse-phase–cation exchange SPE column (Oasis-MCX, Waters) at room temperature. Two hormone fractions were sequentially eluted: (1) fraction A, eluted with methanol containing auxins, ABA, IAA, SA, JA and (2) fraction B, eluted with 0.35-M NH_4_OH in 60% methanol containing cytokinins. Hormone metabolites were analyzed using HPLC (Ultimate 3000, Dionex), coupled to a hybrid triple quadrupole/linear ion trap mass spectrometer (3200 Q TRAP, Applied Biosystems). Quantification of hormones was done using the isotope dilution method with multilevel calibration curves (*R*_2_ >0.99). Data processing was carried out with Analyst 1.5 software (Applied Biosystems). Data are presented as means ± SE. Three biological replicates were analyzed.

### Bioinformatic Analysis of Proteins and Biological Functions of Identified Proteins

Molecular functions of proteins were searched in AgBaseGORetriever (McCarthy et al., 2006) (http://agbase.msstate.edu/cgi-bin/tools/goretriever_select.pl). For Gene Ontology annotation (GO), GOSlimViewer (http://www.agbase.msstate.edu/cgi-bin/tools/goslimviewer_select.pl) was used to characterize general cellular components, biological functions, and biological processes (Ag Base version 2.00; Select GOSlim set: Plant). Only the original identified sequences were used for these analyses.

GOModeler is a tool that enables researchers to conduct hypothesis-based testing of high throughput datasets using the GO (http://agbase.msstate.edu/cgi-bin/tools/GOModeler.cgi). All information necessary for understanding the program processes is covered by Manda et al. (2010). The net effect is a product of quantitative value (logarithm of protein abundance expressed as stress density/control density) with qualitative value (+1, 0, or −1; assessed by GOModeler according to GO annotation of each protein). Program inputs: GOModeler settings used in this article are as follows [for details, refer Urban et al. (2017)]:

**Table:** 

Gene Information Input Type: Uniprot Accession
Expression Values Input Type: Log10 (Treatment/Control)
Species: *Arabidopsis*
Default Effect of Unsigned GO Terms Positive
Default Method for Conflict Resolution for Contradictory GO Terms: Positives
Override

Proteins were sorted into clusters according to their mode of accumulation using Permut Matrix (Caraux and Pinloche, 2005) (version 1.9.4.) on the basis of Z-score standardization of protein density data. In order to draw Venn diagrams, we used “Venn Diagram Plotter,” available on http://omics.pnl.gov/software/venn-diagram-plotter. For data used to characterize “unnamed proteins,” the protein accession versions were blasted to *Arabidopsis* (*taxid: 3701*) in NCBI blastp (https://blast.ncbi.nlm.nih.gov) and manually searched for a gene locus in TAIR (www.arabidopsis.org). Blastp search Database: Non-redundant UniProtKB/SwissProt sequences; Molecule Type: Protein; Update date: 2016/12/11; Number of sequences: 465,342; *Arabidopsis* (*taxid:3701*); and Matrix: BLOSUM62.The mass spectrometry proteomics data have been deposited to the ProteomeXchange Consortium *via* the PRIDE partner repository with the dataset identifier PXD024552.

### Statistical Analysis of Differentially Accumulated Physiological, Biochemical, and Protein Data

Exploratory data analysis (EDA) was used to determine statistically important features of the measured dataset. A combination of statistical tests, together with diagnostics graphs, was used for descriptive statistics (mean, variance, etc.), verification of normality and homogeneity of the data, and detection of the outliers. Linear dependence of the parameters of interest was determined by correlation and regression analyses. For a deeper understanding of the relationship between measured characteristics, principal components analysis (PCA) was used. The same method was also applied to a protein dataset. Diagnostic indicators, such as the Scree plot, loading plot, and the total amount of explained variability, were used to find an optimal model. All statistical tests were computed in STATISTICA ver.12 (StatSoft, Inc.). Cluster analysis of the final protein spot relative abundances has been carried out using Permut Matrix software (Caraux and Pinloche, 2005) (version 1.9.4). For all cluster analyses, the Z-score transformation of data was carried out. Euclidean distances (dissimilarity) and Ward's criteria (rows linkage rule) were used for the analysis. For every Permut Matrix analysis, the highest rows and columns objective functions (R) and a sum of all pairwise distances of neighboring rows or columns (S; shortest path length) were chosen to describe patterns within genotypes and treatments.

## Results and Discussion

Two cultivars of winter oilseed rape differing in their drought-adaptation strategies (detailed description in Supplementary Table 1) were included in this analysis. According to our previous study (Urban et al., 2017), D is a drought-resistant water-saver, and V is drought-susceptible water-spender. The cv. V is considered an early and cv. D as intermediate/late cv. (Urban et al., 2017). Embryos, derived from microspores, were placed in the control and PEG-activated media and harvested after 24 h and after 7 days.

### Biomass Accumulation and Other Physiological Characteristics Revealed Better Adaptation of D to PEG-Infused Media

**Figure 1:**
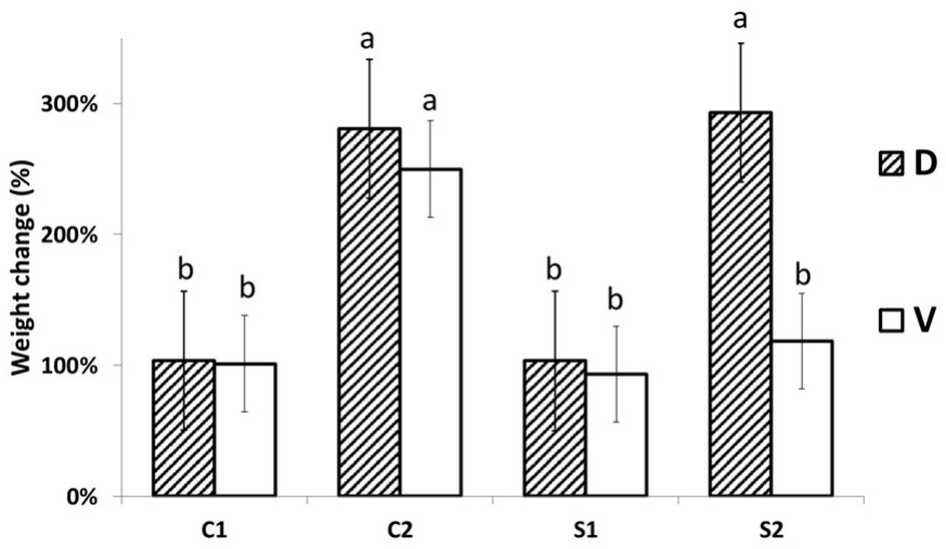
MDE growth changes. MDE relative (%) fresh weight changes (the day after stress +1/day after stress 0) are significantly lower in Viking (V) in comparison to Cadeli (D) only after 7 days of cultivation under stress (S2). Controls and treated samples (C1 and S1, respectively), for the first sampling (24 h after stress); C2, S2 arecontrols and treated samples seven DAS, respectively. Homogeneous groups above the error bars indicate significant changes between cvs. by the Student's *t*-test (*p* < 0.05). Only different letters show significantly different values.

The responses of MDE to steady low osmotically activated plates (Petri dish) were observed after 24 h (acute response) and after 7 days (long-term + recovery response). The MDE fresh weight changes 1 day after stress (DAS; C1 and S1) and 7 DAS (C2 and S2) are shown in Figure 1. The osmotic potential of aqueous PEG 4000 solution (30 g w/v) was measured by vapor pressure deficit osmometer WESCOR Psypro, and its value was −1.05 MPa at 25°C. The osmotic potential of solid media in control conditions was −1.11 MPa at 25°C. This potential changed after it was osmotically activated by PEG solution to −1.55 MPa at 25°C. No statistical difference between MDE biomass accumulation was detected until 7 DAS of PEG treatment, making this system reliable for further use (additionally, the osmotic potential of well-sealed media in Petri dishes without plants did not change even after 3 months; data not shown). Biomass accumulation in PEG-treated D was significantly higher (3-fold) than in the case of V. The increase of biomass in PEG-treated cultivation media agrees with the water-saver behavior of D. The cv. D MDE seems to be better adapted and then able to acclimate and grow under increased osmotic potential (or osmolality). We can only hypothesize whether this reflects the observed field phenotype (published earlier) or not. The D biomass increase is similar to its growth under control conditions. V slightly decreased its MDE weight (not significant; Figure 1) after 7 days in the control medium. The other explanation may be a higher and rapid accumulation of osmolytes in D vs. V, which further stabilizes MDE cytosol against PEG-driven dehydration.

The observed MDE growth results agree with previous reports and show that even only data of MDE growth on osmotically activated media can be used as a screening tool to select resistant cultivars. In addition, the stability of the system suggests the non-significant artifact effect of cultivation technique on growth characteristics (Supplementary Figure 1). However, further study needs to be conducted to verify the stability of MDE derived from different cultivars regressed to their actual field-observed phenotypes.

### Proteomic Analysis of V Embryos Showed a High Number of Accessions Changed in Stress/Defense-Related Processes

Two genotypes of winter oilseed rape cultivars (D and V) exhibit contrasting levels of drought resistance, and two different treatments (control and PEG-induced drought simulation) were compared after 7 DAS in the 2D-DIGE proteomic experiment. Despite the same age of embryos, PEG-treated MDE of both cvs. was slightly dwarfish (likely because of low water tissue content upon osmotic; Supplementary Figure 1), however, without discoloration (suggesting no mineral deficiency).

**Figure 2:**
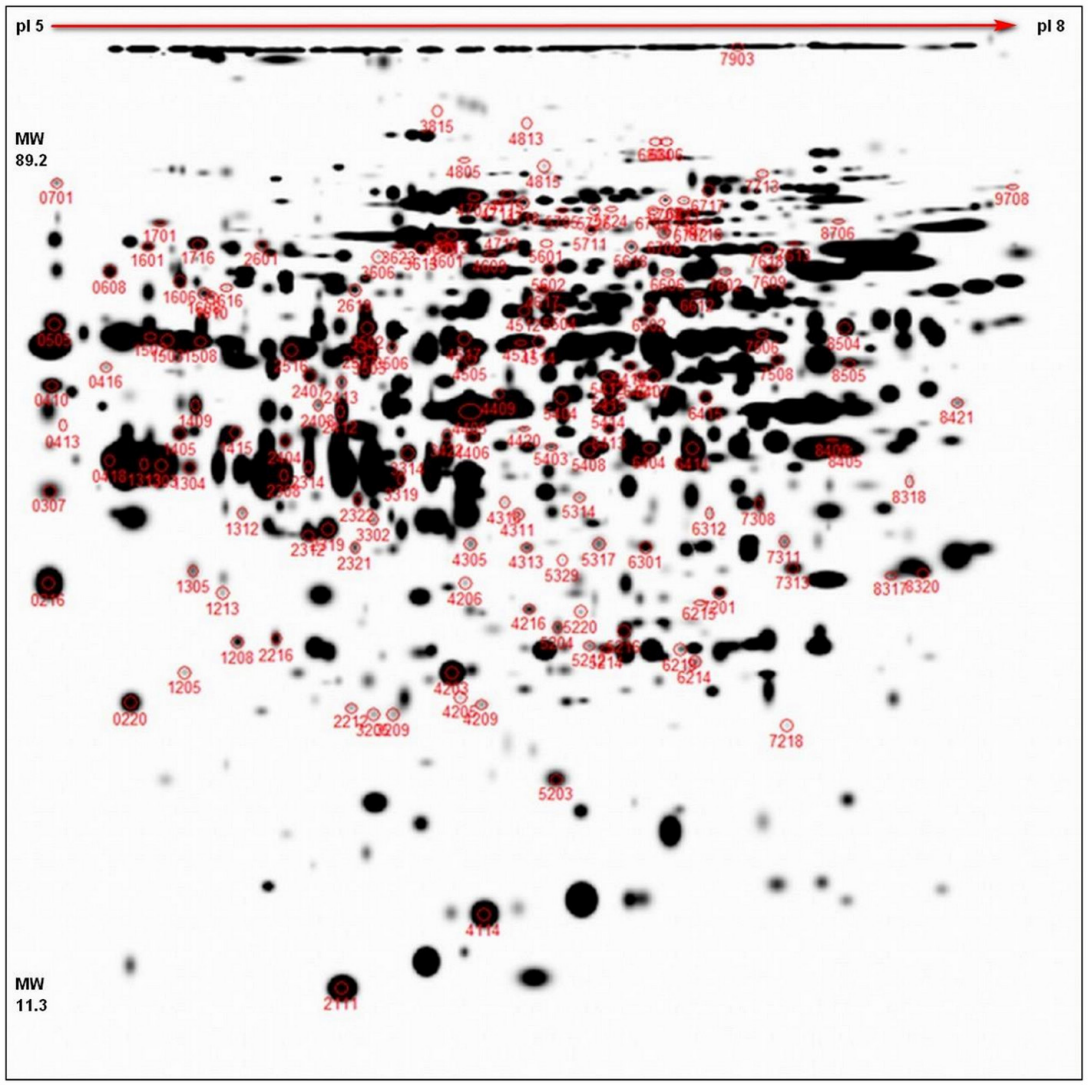
2D-DIGE representative proteome map. Representative proteome map of total MDE proteins of winter oilseed rape, separated by 2D-DIGE. At least two times up- or down-accumulated proteins (*p* < 0.05, 11 cm IPG strip, pI 5–8, 1 mm thick gel) are marked with the corresponding spot numbers. In total, 156 spots chosen for cutting and identification are shown in red circles.

**Figure 3:**
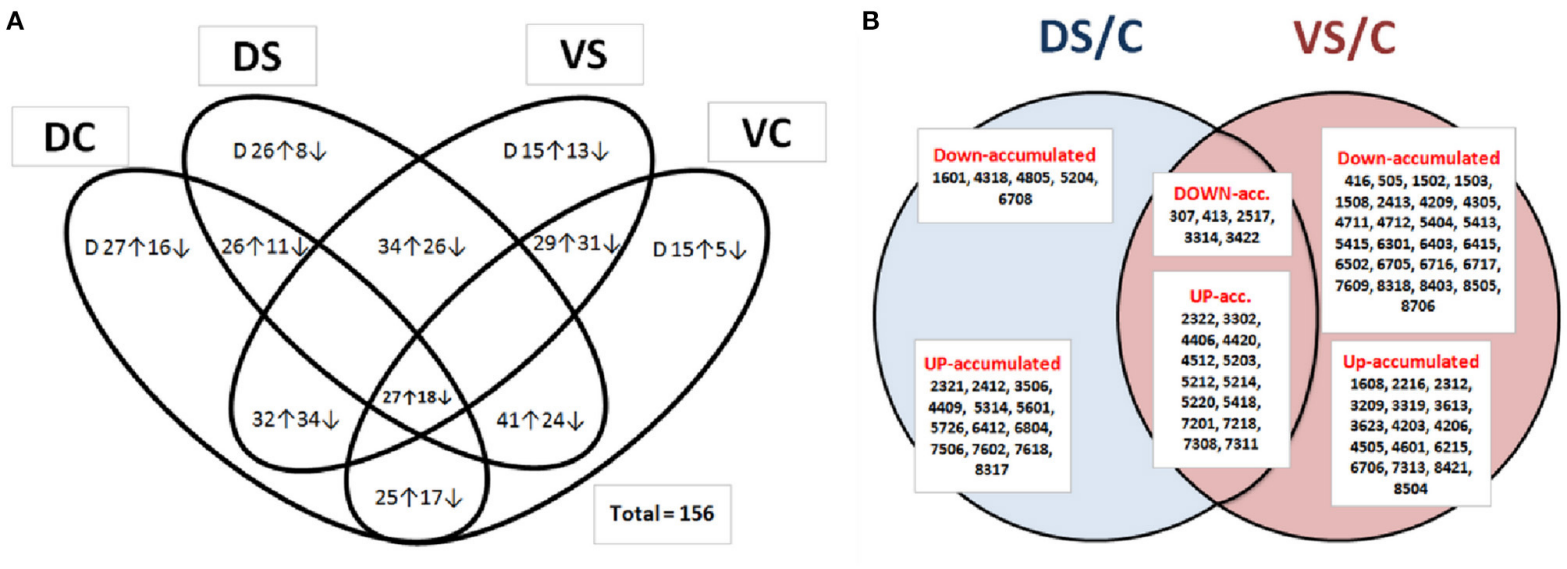
Venn diagram of protein abundances. Venn diagram of identified and non-identified spots **(A)**, showing all differentially abundant protein spots (at least 2-fold change at *p* < 0.05 and SD ≤ 0.5; *n* = 156), revealing differential abundance between PEG-treated and control samples (DC, Cadeli control; DS, Cadeli stress; VC, Viking control; VS, Viking stress). Venn diagram **(B)** showing SSPs of unique identified protein spots (at least ± 2-fold change at *p* < 0.05 and SD ≤ 0.5; *n* = 63), revealing differential abundances between PEG-treated and control samples among genotypes. DS/C, Cadeli-treated/control, 18 SSPs; VS/C, Viking-treated/control, 41 SSPs and its union (19 SSPs). ↑ The arrow means increased abundance with respect to a ratio, while ↓ means decreased abundance with respect to a ratio. Letter “D” in Figure 3A inside ovals means a change of protein spot density just over the background of DIGE gels (at least 2-fold change; *p* < 0.05 and SD ≤ 0.5) because they do not have a comparison to other treatment.

The representative 2D-DIGE gel of MDE with highlighted 156 spots, showing protein resolution, is shown in Figure 2. All normalized protein spots (894 spots) and these chosen for identification (156) protein spots across the gels were included in two separate PCAs to identify sample outliers and to group samples from different stages of treatment for each cultivar (SI 1). Two PCAs were prepared: (1) analysis based on all raw protein abundances of all individual gels (Supplementary Figure 2) and (2) analysis based on averaged values of protein abundances of individual samples (DC, DS, VC, and VS; Supplementary Figure 3). PCA based on all values (Supplementary Figure 2) distinguished both genotypes in all factors 1–3 and may explain almost 33% of data variability. PCA based on averages of abundances (Supplementary Figure 3) distinguishes between controls samples from treated samples between cultivars, despite the fact DC+VS and DS+VC were projected closer to each other. This result is caused by the fact that these groups share high numbers of similarly oriented (down- or up-accumulated) proteins (refer Figure 3) and it is supported also by PermutMarix clustering analysis. Factors 1–2-based projection of Supplementary Figure 3 data may explain almost 66% of data variability. The protein spots belonging to 156 chosen spots are placed in the center of the plots of the PCA, which revealed that spots exhibit contradictory accumulation patterns within genotypes (and ergo the mean is centered close to zero).

**Figure 4:**
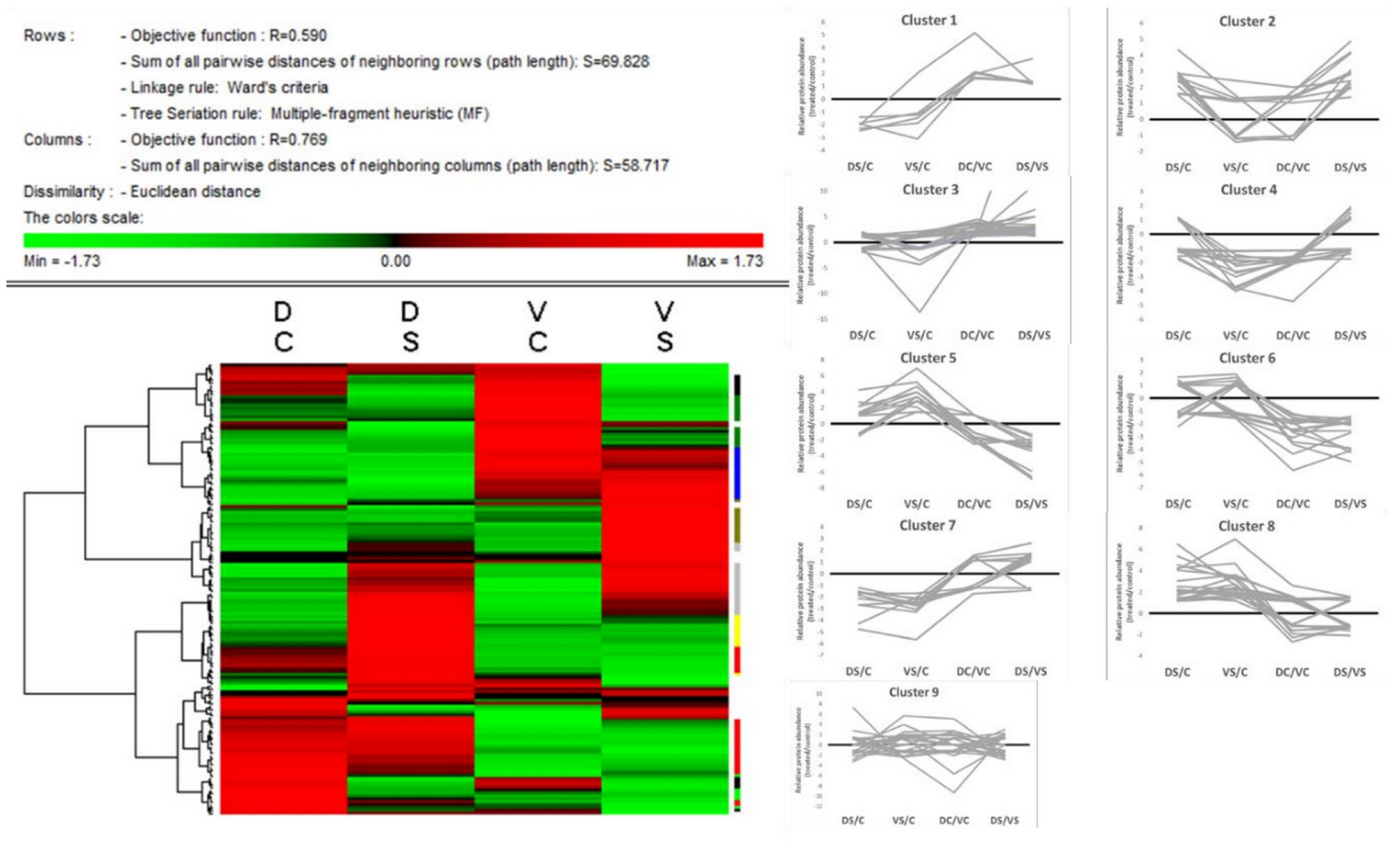
Heat map + protein clusters. The compacted version of the heat map (Permut Matrix 1.9.3) of all 156 protein spot abundances, clustered according to the row Z-score values using Euclidean distance and Ward's minimum criteria, calculated from protein spot abundances. The red values mean higher protein spot abundance in the samples; the green values mean lower protein abundance in the sample according to the variant (DC, Cadeli control; DS, Cadeli stress; VC, Viking control; VS, Viking stress). On the right side, the tiny color marks show nine different clusters, revealing information about the protein accumulation patterns. Clusters: 1, light green, higher in DC; 2, light yellow, higher in DS; 3, red, higher in both DC and DS; 4, green, higher in VC; 5, dark yellow, higher in VS; 6, blue, higher in both VC and VS; 7, black, higher in both controls; 8, gray, higher in both treated samples; and 9, white, miscellaneous patterns.

The heat map (Figure 4) was created on the basis of standardized protein abundance of 156 chosen protein spots (standardization by Z-score values) in Permut Matrix. All differently accumulated spots (156 spots) showed clustering into nine main clusters according to their accumulation pattern in controls and treated genotypes (Supplementary Table 2; or graphs in SI 1). These nine clusters were divided according to genotypes and treatments as follows: Cluster 1, proteins accumulated mainly in DC; cluster 2, proteins accumulated mainly in DS; cluster 3, proteins with generally higher abundance in cv. D; cluster 4, proteins accumulated in VC; cluster 5, proteins mainly accumulated in VS; cluster 6, proteins with generally higher abundance in cv. V; cluster 7, proteins accumulated mainly in controls; cluster 8, proteins accumulated mainly in treated samples; and cluster 9, proteins with mixed patterns of accumulation.

Some proteins (e.g., cobalamin-independent methionine synthase, glyceraldehyde-3-phosphate dehydrogenase) have been found in more protein spots; however, they belong to different accumulation clusters. It refers to one of the main advantages of the gel/based method—the possibility to visualize and quantify different gene products and/or posttranslational modifications of the same protein that could have different or opposite accumulation under the same growth condition.

Generally, cv. D showed a high number of proteins changed in the amino acid (AA), protein, and energy metabolism (protein clusters 1 and 3) category. Cv. V showed a high number of proteins changed in AA, protein, and energy metabolism and stress/defense-related processes (clusters 1, 3, and 5). Interestingly, both treated cvs. (cluster 8) showed an enhanced accumulation of proteins involved in energy metabolism and redox homeostasis, ROS, and signaling (clusters 3 and 4).

According to Venn diagrams (Figure 3A; additional graphs in SI 2b, 2c), D showed the highest number of unique spots differently up- or down-accumulated in controls and a treated variant. On the other side, cv. V showed a high number of genotype-related variants. This result can partially be explained by different growth rates in control and PEG-treated conditions (Figure 1). In contrast to the data shown in Figure 3A, when the S/C protein ratio was used in a treatment-based variant (Figure 3B), significantly, more proteins were down-accumulated in the VS/VC ratio. In DS/C, only 5 and 13 were down- and up-accumulated, respectively. For VS/C, 25 and 16 were down- and up-accumulated, respectively. This result showed that cv. V was more influenced by changes in cultivation conditions and that its homeostasis was disturbed. In conclusion, the protein abundance is likely the cause of the different growth rates (genotype behavior described in Supplementary Table 1), not a consequence.

Eleven spots were not sufficiently identified, probably due to a lack of sequence similarity or low abundance; nevertheless, these spots were included in Permut Matrix clusters and PCA analysis. The table of identified proteins (SI 2a) contains all 63 successfully identified spots. The search for identified protein spots with large changes in protein abundance (more than ± 3-fold; *p* < 0.05) has revealed seven protein spots (aspartate aminotransferase, AT2G47510-fumarate hydratase 1, rubisco, peroxiredoxin antioxidant, peroxidase 12, jasmonate inducible protein, and elongation factor EF-2-like protein). Highly accumulated proteins belong mostly to protein and energy metabolism, and stress/defense-related proteins.

### Functional Categories of Drought-Responsive Proteins Highlighted Eight Functional Groups

**Figure 5:**
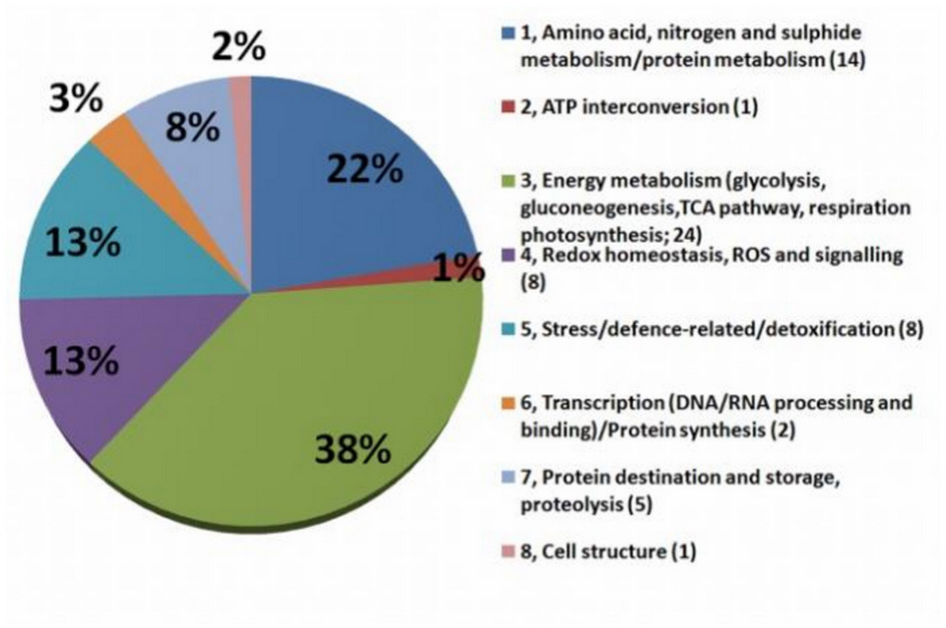
Graph of functional groups. The functional classification of MDE proteins differentially accumulated after 7 days after stress (*n* = 65). The numbers in brackets are numbers of proteins involved in each process.

Detailed information about protein identification *via* the NCBI database is presented in SI 3. The biological functions of individually differently accumulated proteins were determined by GO Retriever output in the domain of biological processes according to their NCBI Accession version (SI 4). To investigate the functional and biological process-based identity of the individually identified differentially accumulated proteins (DAP), the 63 identified spots were categorized into eight groups (Figure 5; details in SI 2a) based on their putative biological processes: (1) Amino acid, nitrogen, and sugars metabolism/protein metabolism (13 DAP); (2) ATP interconversion (1 DAP); (3) energy metabolism (glycolysis, gluconeogenesis, TCA pathway, respiration, and photosynthesis) (24 DAP); (4) Redox homeostasis, ROS, and signaling (7 DAP); (5) stress/defense-related/detoxification (8 DAP); (6) transcription (DNA/RNA processing and binding)/protein synthesis (2 DAP); (7) protein destination and storage, proteolysis (5 DAP); (8) cell structure (1 DAP).

**Figure 6:**
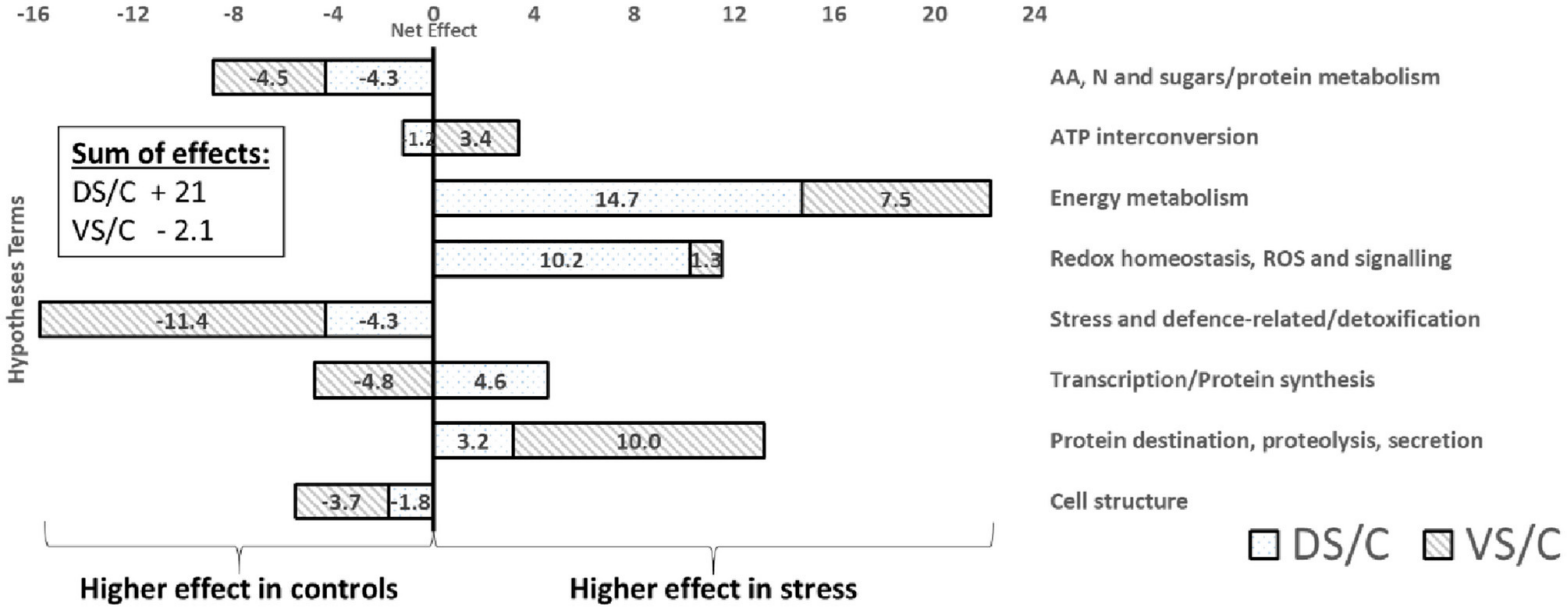
GOModeler output. The GOModeler-based graphical summary of proteome net effects for each of eight chosen hypotheses terms (right side), showing net effects on treated protein accumulation (protein abundance change). The net effect is a product of quantitative value (logarithm of protein abundance expressed as stress density/control density) with qualitative value (+1, 0, or −1; assessed by GOModeler according to GO annotation of each protein). In the corner, the sum of all net effects for individual combinations is calculated. A positive value (right side of the graph) means higher protein net effect in treated plants; negative values mean higher net effect importance in controls.

GOModeler output of functional groups (Figure 6) reveals groups with higher estimated effects (net effect) in controls (e.g., AA, sugar/protein metabolism; stress/defense-related; and cell structure) and higher importance of energy metabolism, ROS/redox, and protein destination groups in treated plants. Interestingly, when cultivars were GOModeler compared (Supplementary Figure 4), higher importance of AA, sugar/protein metabolism, ATP, stress/defense-related with protein destination for V was observed. A significantly higher net effect in D was observed in energy metabolism, ROS/redox, and protein synthesis groups.

According to these eight functional groups, we designate these groups most affected by PEG-related osmotic stress in studied winter oilseed rape MDEs. Differences found in the numbers of proteins responsible for the individual biological processes influenced by osmotic stress indicate the existence of diverse response strategies to osmotically-driven drought between the selected contrasting genotypes. This conclusion was also proved in drought-related differential leaf proteomics of four cvs. in Urban et al. (2017).

Generally, a significant decrease in the accumulation of proteins could be explained by the reduction of a growth rate and/or different development of strategy under osmotic stress conditions.

### Functional Groups Regarding *B. napus* MDE Specific Response to Osmotic Stress

All differentially abundant proteins, which were successfully identified, and their distribution into functional groups with detailed information are available in SI 2a, with details from NCBI identification in SI 3.

#### Amino Acid, Nitrogen, and Sugars Metabolism/Protein Metabolism

The proteins in this group are involved in the chemical reactions and pathways concerning organic or inorganic compounds that contain nitrogen and sulfide interconversion with sugars and protein metabolism. Sugars play a central regulatory role in many vital processes besides serving the energetic function and are considered important signals that regulate plant metabolism and development (Kosova et al., 2015). This group represents the second biggest part of an identified protein. In the *Brassicaceae* family, the nitrogen and sulfide compounds are important from point of view of metabolism (thiols, glucosinolates, brassinosteroids) and biotic and/or abiotic stress adaptation (Sehrawat and Deswal, 2014). *B. napus* genotypes with high sulfur- and nitrogen-use efficiency are more resistant to PEG-induced osmotic stress (Limami et al., 2008). This functional category includes proteins that are significantly down-accumulated in DC vs. VC (e.g., isocitrate dehydrogenase, glutamine synthase, sucrose synthase, and glyoxalase). Some proteins are only accumulated in treated D vs. V (aspartate aminotransferase, cobalamin-independent methionine synthase, and nodulin).

#### Energy Metabolism (Glycolysis, Gluconeogenesis, TCA Pathway, Respiration, and Photosynthesis)

To maintain sufficient energy and balanced carbohydrate production belong to one of the most important pathways in all plants as sessile organisms. In contrast to Urban et al. (2017), energetic metabolism-related proteins and photosynthesis-related ones were joined together into one group. The ability of plants to adapt and/or acclimate to adverse environments is related to the plasticity and resilience of photosynthesis, which, in combination with other processes, determines plant growth and development (Abreu et al., 2013). This group represents the biggest part of identified proteins (Figure 5). Some carbon/nitrogen metabolism-related proteins identified (e.g., malate dehydrogenase), show increased energy demand as well as enhanced cellular activities in the root tissues of rapeseed upon drought (Mohammadi et al., 2012).

Most protein spots were up-accumulated in cv. D in comparison with cv. V. Also, between treatments (S/C of individual genotypes), most proteins were significantly changed in cv. V (e.g., glyoxysomal beta-ketoacyl-thiolyase, fumarate hydratase 1, FBA, phosphoenolpyruvate carboxykinase). These changes support the link between the growth (Figure 1) and energy metabolism proteins. cv. D grew significantly more in treated conditions than cv. V, probably because of its enhanced levels of proteins involved in energy metabolism under stress.

Unfortunately, no clear consensus has been achieved that would reveal stress-resistant genotypes according to their changes in energy metabolism-related proteins. More DAPs were accumulated in cv. D (both in controls and treated samples). Nevertheless, this is only a quantitative point of view; several proteins play an important qualitative role, too.

#### Redox Homeostasis, ROS, and Signaling

Together with stress/defense-related proteins, this category contains the third most abundant protein pool. The cellular redox homeostasis is significantly affected by the stress-induced production of ROS, which also serves as a signal for the synthesis of defense enzymes and other antioxidant systems. Together with the photosynthesis and stress proteins, the redox homeostasis and signaling are likely to integrate all stresses into the cellular response with a stress-adaptive program (Fernandez and Strand, 2008). Spots found in this study show that the antioxidant system and ROS production play a crucial role in MDE resistance and should be further examined to contribute to the selection of adaptable rapeseeds. An increase in several ROS scavenging enzymes was reported practically in all proteomic studies dealing with plant stress responses, as the imbalance in energy metabolism during stress treatments is associated with an enhanced risk of oxidative stress (Kosova et al., 2011). Proteins in this category showed up-accumulation in both treated cvs. Interestingly, several proteins showed higher accumulation in controls of V in comparison to lower accumulation in D (e.g., alcohol dehydrogenase class III, S-nitrosoglutathione reductase, peroxiredoxin antioxidant, etc.). A similar trend was clearly visible in the leaf proteome study of cv. V in comparison with other cvs. (Urban et al., 2017) but in contrast to drought-related transcriptomic study, where higher ROS were joined to tolerant rapeseed genotypes (Schiessl et al., 2020).

#### Stress and Defense-Related Proteins

Half of the proteins in this group showed down-accumulation in treated vs. control samples. Only MLP-like protein 329 (SSP 4114) was accumulated in treated samples in both cvs. cv. D generally showed a lower accumulation of stress and defense-related proteins than cv. V. In the DS/VS ratio, only catalase, and ABA-modulated tyrosine-phosphorylated proteins were more accumulated.

#### Transcription, Protein Synthesis/Protein Storage/Cell Structure

This is an artificial category, jointing the remaining three small functional groups together (6–8). Proteins in DNA/RNA processing and binding/protein synthesis showed higher accumulation in treated D vs. treated V. The cruciferin cru 2/3 subunit significantly increased in both treated cvs., while tubulin decreased.

### High ROS, Protein Turnover, and Cell Trafficking System With Available Energy-Related Pathways Support D Resistance

The highest number of proteins accumulated significantly in D vs. V (clusters 1–3; 9) belongs to energy metabolism, redox homeostasis + signaling, transcription, protein destination, storage, and proteolysis. Cv. D showed effective energy-related pathways, higher sensing of ROS-related changes in cell compartments, higher protein turnover, and/or synthesis and, also, an increase in cell trafficking system. Selected proteins with a changed abundance in D are listed.

Aspartate biosynthesis is mediated by the enzyme aspartate aminotransferase (de la Torre et al., 2014a) (SSP 1405, 2404); both its cytosolic and plastid forms play a central role in nitrogen metabolism and its storage (de la Torre et al., 2014b). The enzyme activity increases after infection with a necrotrophic pathogen (Brauc et al., 2011). One gene product (isoform) of aspartate aminotransferase is accumulated in cv. D (SSP 2404), the other in cv. V (SSP 1405). In the chloroplasts and in non-green plastids, aspartate serves as the precursor for the biosynthesis of different amino acids and derived metabolites that play distinct and important roles in the regulation of plant growth, reproduction, development, or defense. This protein probably plays a dual role in both somatic embryogenesis and stress responses, as proposed by Almeida et al. (2012).

Proteins fluG-like (SSP 5705, 5724, and 5726) were searched by Blastp and proved similar to nodulin/glutamate-ammonia ligase-like proteins (NodGS). NodGSs belong to the glutamine synthetase family. Recent studies highlight the importance of nodulin-like proteins for the transport of nutrients (especially nitrogen), solutes, amino acids, or hormones and for major aspects of plant development (Denance et al., 2014). Some of the nodulins showed aquaporin activity (Katsuhara et al., 2014), facilitating water, hydrogen peroxide, and even arsenite transports out of the cytosol. Doskocilova et al. (2011) pointed the role for NodGS in root morphogenesis and microbial elicitation. However, the role of NodGS in abiotic stress response is still unknown. NodGS proteins (SSP 5705, 5724) were accumulated significantly more in DS (in comparison to VS). SSP 5726 was highly accumulated in both treated cultivars in comparison with its controls.

AT4g37510/F6G17_160 (SSP 5601; NDH-1) is a subunit of the mitochondrial membrane respiratory chain NADH dehydrogenase (Complex I) and functions in the transfer of electrons from NADH to the respiratory chain (Klodmann and Braun, 2011). NDH-1 belongs to Quinone reductases (QRs), which are flavoproteins that protect organisms from oxidative stress. The function of plant QRs has not been as yet addressed *in vivo* despite biochemical evidence for their involvement in redox reactions (Heyno et al., 2013).

The AT2G47510 (SSP 416, FUM1) is recognized as fumarase 1, which is a mitochondrial-localized protein and plays an important role in the tricarboxylic acid cycle (TCA). FUM1 was down-accumulated in both treated samples; however, it was generally much more accumulated in cv. D in contrast to V.

Among the other energy metabolism-related proteins with significantly higher accumulation in treated samples of cv. D (DS/DC) is UDP-glucose 6-dehydrogenase (SSP 3506). UDP-glucose dehydrogenase (UGD) plays a key role in the nucleotide sugar biosynthetic pathway, as its product UDP-glucuronic acid is the common precursor for many sugar residues found in the cell wall (Klinghammer and Tenhaken, 2007). The importance of UDP GlcA for plant primary cell wall formation was also shown by Reboul et al. (2011).

Two proteins from the redox homeostasis, ROS, and signaling group were accumulated only in DS: S-nitrosoglutathione reductase (SSP 2412, GSNOR) and mitogen-activated protein kinase 4 (SSP 5403, MAPK4). Nitric oxide (NO) may react with glutathione (GSH) to form GSNO, which is considered the main reservoir of NO in cells (Silveira et al., 2016). The redox-active molecule NO is known to modulate plant responses to stressful conditions, plant immunity (Thalineau et al., 2016), cross talk in salt resistance (Fatma et al., 2016), photosynthetic apparatus protection and to improve shoot and root growth upon drought in sugarcane (Silveira et al., 2016), etc.

The MAPK is a generally important factor in the regulation of signal transduction in response to biotic and abiotic stresses (Yanagawa et al., 2016). Gawronski et al. (2014) and others concluded that MAPK4 is a complex regulator of chloroplast retrograde signaling related to photosynthesis, growth, and immune defense in *Arabidopsis*. MAPK4 is also recognized as a salicylic acid-independent regulator of growth (Gawronski et al., 2014), expression of brassinosteroid-related genes in rice (Duan et al., 2014), and general abiotic/biotic stress response in barley (Abass and Morris, 2013).

The peroxiredoxin antioxidant (SSP 5214) and hydroxyacylglutathione hydrolase 3 (SSP 5220) were significantly accumulated in both treated cvs. This result is in accordance with Urban et al. (2017). Peroxiredoxins (Prx) are known to play an important role in combating the reactive oxygen species generated at the level of electron transport activities in plants exposed to different types of biotic and abiotic stresses. Kim et al. (2012) suggested that in Brassicaceae Prx isotypes play time- and issues-specific roles in the cells, but that they also cooperate with each other to protect the plants. Hydroxyacylglutathione hydrolase 3 (SSP 5220; ETHE) is also called “persulfide dioxygenase” and is located in mitochondria. ETHE catalyzes the oxidation of persulfides in the mitochondrial matrix and is essential for early embryo development in *Arabidopsis* (Holdorf et al., 2012; Krussel et al., 2014).

Surprisingly, no protein from the stress-related group was significantly up-accumulated in DS. Ascorbate peroxidase (SSP 5204; APX) exhibited an enhanced DC/VC ratio, and catalase (SSP 1502), with ABA-modulated tyrosine-phosphorylated protein (SSP 4216), was elevated in stressed D (high DS/VS ratio). APX was found to be specifically required for the resistance of *Arabidopsis* plants to drought and heat stress combination (Mittler and Blumwald, 2010). In the thylakoid lumen, APX is essential for photo protection as a cofactor of violaxanthin de-epoxidase, the key enzyme in non-photochemical quenching. It has to be mentioned that H_2_O_2_ plays a signaling function in the modulation of plant phenotype (Huener et al., 2012); therefore, the APX is a powerful part of a complex response to any biotic and/or abiotic stresses.

Also, transcriptional factor Pur ALPHA-1 (SSP 5314; PurA) involved in RNA processing showed accumulation in DS. PurA is a single-stranded DNA-binding protein that plays a role in cell growth and differentiation by modulating both transcriptional and translational controls of gene expression.

SSP 4711 was blasted to elongation factor EF-2-like protein LOS1 (SSP 4711; EF2). This spot was significantly reduced in VS and accumulated in DS. The DS/VS ratio was more than 11. LOS1 encodes a translation elongation factor 2-like protein that is involved in cold-induced translation; however, more importantly, LOS means “low expression of osmotically responsive genes.”

Three protein spots belonged to protein destination and a storage group: 26S proteasome ATPase subunit, cruciferin cru2/3 subunit, and Clp ATPase. 26S proteasome ATPase subunit (SSP 2407) belongs to AAA+ (ATPases associated with a wide variety of cellular activities). This superfamily represents an ancient group of ATPases. Members of the AAA+ ATPases function as molecular chaperons, ATPase subunits of proteases, helicases, or nucleic-acid stimulated ATPases. This protein showed a significant ratio in DC/VC.

Clp ATPase (SSP 6710) belongs also to AAA+. This protein is sometimes called “HSP93-III” and is involved in protein import into chloroplast stroma. This protein showed an increased value in the DS/VS ratio.

### Higher Abundance in ATP Interconversion Pathways With Stress/Defense Proteins Shows the Higher Vulnerability of V

The proteins exhibiting an enhanced relative abundance in cv. V (clusters 4–6; 9) belong to four functional groups: AA, nitrogen, and protein metabolism; ATP interconversion; stress and defense-related/detoxification; and cell structure. This fact supports the idea about the higher need for ATP and nutrient (re)utilization, deeper stress impact, and increased stress-related cell structure changes. This is also in agreement with slower MDE growth (Figure 1). Selected proteins with significant changes in V are described below.

Glutamine synthetase precursor (SSP 5408; EC 6.3.1.2; GS) decreased in VS and also in relation to DC/VC. GS is an ATP-dependent plastid enzyme that plays an essential role in the metabolism of nitrogen and in photorespiration where it is a key enzyme. According to studies (Doskocilova et al., 2011; Orsel et al., 2014), plants with low GS isoform 2 have reduced capacity for photorespiration and decreased resistance to light stress, so they are photo inhibited more severely by high light compared with control plants. GS accumulation can directly protect plants from drought-related photo-inhibition, so D could probably photo-acclimate better because of the lower decrease of GS in treated conditions.

**Figure 7:**
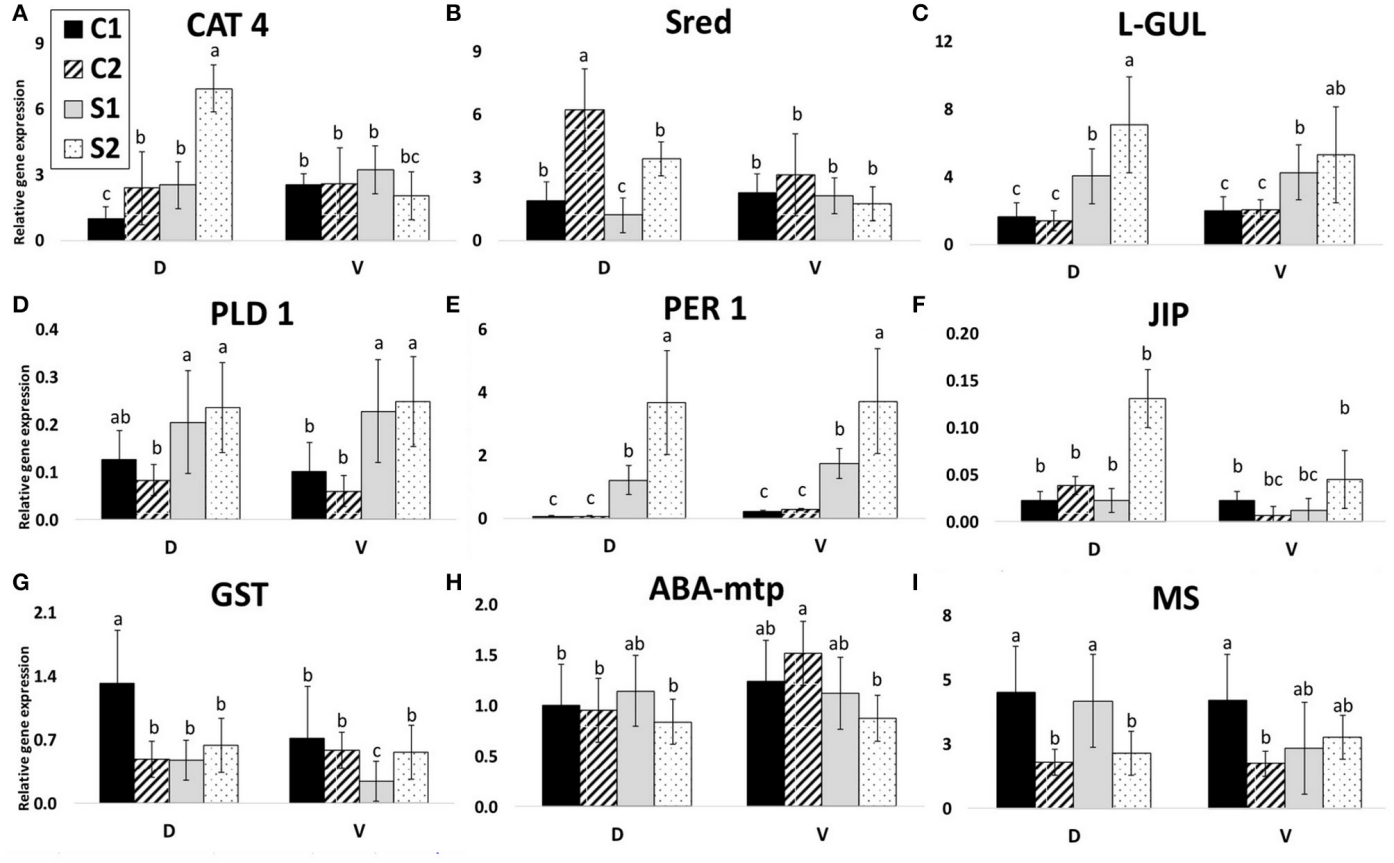
Gene expressions. Relative gene expressions of nine selected genes according to protein identification. C1, control after 24 h after transfer to solid medium; S1, treated samples after 24 h after transfer; C2, control after 7 days after transfer; and S2, treated samples after 7 days after transfer. Gene expression was calculated according to the 2^−Δ*ΔCT*^ method. Means with the same letter within each gene are not significantly different at 5% probability. Error bars denote three technical replications. **(A)** CAT 4, catalase; **(B)** Sred, sultite reductase; **(C)** L-GUL, putative lactoylglutathione lyase; **(D)** PLD 1, phospholipase D alpha 1; **(E)** PER 1, peroxiredoxin antioxidant; **(F)** JIP, jasmonate inducible protein; **(G)** GST, glutathione S-transferase; **(H)** ABA-mtp, ABA-modulated tyrosine-phosphorylated protein; **(I)** MS, 5-methyltetrahydropteroyltriglutamate-homocysteine methyltransferase.

Glyoxalases are known to be differentially regulated under stress conditions, and their overexpression in plants confers resistance to multiple abiotic stresses (Kaur et al., 2014). The glyoxalase system is a set of at least two enzymes (glyoxalases 1 and 2) that carry out the glutathione-dependent detoxification of methylglyoxal and the other reactive aldehydes that are produced by cellular metabolism even under optimal conditions. Putative lactoylglutathione lyase (synonym: glyoxalase 1; SSP 7201) in both treated genotypes was significantly accumulated, as also shown in Urban et al. (2017). The same pattern is shown in the RT-qPCR result (Figure 7C).

ChloroplastatpA gene product, also called “NADH dehydrogenase” (SSP 8504; ATPA), produces ATP from ADP in the presence of a proton gradient across the membrane. ATP production is significantly higher in VS (VS/VC ratio) while no change was observed in DS/DC. This protein was accumulated in treated V samples and was highly suppressed in DS in comparison to VS (−6.6-fold), which suggests a higher energy need of V. This accumulation pattern is contradictory to Urban et al. (2017).

In the energy-related protein category, glyceraldehyde-3-phosphate dehydrogenase (SSP 418) and chloroplast beta-carbonic anhydrase like 1 (SSP 1208; CA1), together with malate dehydrogenase 2 (SSP 2312), F21D18.28 (blasted pyridine nucleotide-disulfide oxidoreductase or dihydrolipoamide dehydrogenase; SSP 3502; PYROXD), and fructose-bisphosphate aldolase (SSP 3319) were uniquely accumulated in VS. CAs are ubiquitous enzymes involved in fundamental processes like photosynthesis, respiration, pH homeostasis, and ion transport. CAs proteins are involved in the CO_2_ signaling pathway, which controls gas exchange between plants and the atmosphere by modulating stomatal movements (Ferreira et al., 2008; Rowlett, 2010). CA promotes water use efficiency by influencing the internal conductance (Warren, 2008; Cui et al., 2012) and is phosphorylated, which has been found important for drought-tolerant rapeseeds (Wang et al., 2016).

Cobalamin-independent methionine synthases (SSP 3619 and 3713; MetE) were found to have mixed accumulation. The predicted function of the cobalamin-independent methionine synthase isozyme is closely related to ethylene biosynthesis (Wang et al., 2014) and then in stress-related signaling. SSP 3619 showed highly significant accumulation in VC; SSP 3713 was accumulated in treated D. Each MetE, however, belongs to different genes (SSP3713 is likely connected to different phenotypes of D).

Phosphoglucomutase 1 (SSP 5602, PGM1) catalyzes the bidirectional interconversion of glucose-1-phosphate (G-1-P) and glucose-6-phosphate (G-6-P) *via* a glucose 1,6-diphosphate intermediate. PGM1 shows higher accumulation in both VC and VS.

MLP-like protein 329 (SSP 4114, NP_565265.1; MLP329) is a pathogenesis-related protein with still no clear function within plants and predicted location in the nucleus and/or chloroplast (Cerny et al., 2013). In both cvs., MPL329 was increased in treated conditions. Interestingly, this protein was accumulated more than 3-fold than in D. Its possible role in cytokinin signaling was mentioned in Cerny et al. (2013) and is discussed in the hormonal profile below.

Glutathione S-transferase (SSP 4203; gi|87294807; GST) is a cytosolic dimeric protein involved in cellular detoxification by catalyzing the conjugation of glutathione (GSH) with a wide range of endogenous and xenobiotic-alkylating agents. GST showed increased abundance only in VS (in opposite to gene expression in Figure 7G). GST is a part of the plant protection mechanisms against toxic oxygen intermediates, together with superoxide dismutases, catalases, ascorbate peroxidases, and glutathione peroxidases (Abreu et al., 2013). Other results suggest that a decrease of the glutathione redox status during embryo development may represent a metabolic switch needed for the elevation of the endogenous levels of ABA contents, which is required for successful completion of the developmental program (Belmonte et al., 2006).

Jasmonate inducible protein (SSP 6717) containing a jacalin-like lectin domain is a lectin. Its accumulation is significantly lower in VS in comparison to VC, which is, however, in comparison with DC 4.7 higher (also supported by gene expression analysis in Figure 7F). Jacalin-like lectins have sugar-binding protein domains mostly found in plants, e.g., salt stress-induced in rice (Zhang, 2014) or wheat cold-regulated (Kosova et al., 2013). More importantly, lectins are involved in the regulation of plant development (e.g., the transition of SAM from vegetative to the reproductive stage).

Cruciferin cru 2/3 (SSP 5203; CRU) is a storage protein (also called “11S globulin”) localized on a rough endoplasmic reticulum. CRU principal function appears to be the major nitrogen source for developing plants. Cruciferins can be classified on the basis of their structure into different families. This family is a member of the “cupin” superfamily on the basis of its conserved barrel domain. Gabrisova et al. (2016) showed that an increased abundance of cupin fragments in radium-contaminated flax contributed to growth and reproduction. CRU showed low relative accumulation in DC and high in VS. However, both cvs. showed significant CRU up-accumulation upon stress.

### Phospholipases and Rubisco Precursor—Proteins Accumulated Mainly in Controls or Treated Samples Support Results From Hormonal Profiling

Proteins in cluster 7 did not show differences between treatments but differential accumulation in controls or upon stress (cluster 8). However, some of them show a mixed pattern of their gene products (isoforms; cluster 9). Therefore, they were similarly up- or down-accumulated despite treatments in both cvs. However, to understand all proteome changes and have deeper insight, these data are discussed to share the complexity of the MDE answer to PEG-related osmotic stress.

Rubisco ssu precursor (SSP 2111) showed a very high increase in DS/DC (seven times) and a significant decrease in the VS/VC ratio. However, VC showed nine times the higher accumulation of Rubisco SSU precursor (ssp 2111) than in DC but, interestingly, higher accumulation in DS in contrast to VS.

Fructose-bisphosphate aldolase 3 (SSP 2322 and 3319; EC 4.1.2.13; FBA) catalyzes the reversible cleavage of fructose-1,6-bisphosphate to glyceraldehyde 3-phosphate and dihydroxyacetone phosphate. Glycolysis uses this forward reaction, while gluconeogenesis and the Calvin cycle, which are anabolic pathways, use the reverse reaction. However, these reactions are not possible under energy deficit. The level of FBA was found to decline under salt stress in most of the plant species studied [reviewed by Abreu et al. (2013)]. Yin et al. (2008) found FBA important in maintaining plant physiological functions during wound response in leaves of *B. napus*. Aldolase has also been implicated in many non-catalytic functions based upon its binding affinity for multiple other proteins, including tubulin and phospholipase D, which were found in this study (decreased in both treated samples).

Malate dehydrogenase (SSP 2312 and 2319; EC 1.1.1.37; MDH) reversibly catalyzes the oxidation of malate to oxaloacetate using the reduction of NAD+ to NADH. This reaction is a part of many metabolic pathways, including the citric acid cycle and gluconeogenesis. MDH showed mixed abundance in stressed plants, which cannot support an idea about its important role in water transport and the need for NADPH reduction power, as was postulated in our previous study (Urban et al., 2017).

**Figure 8:**
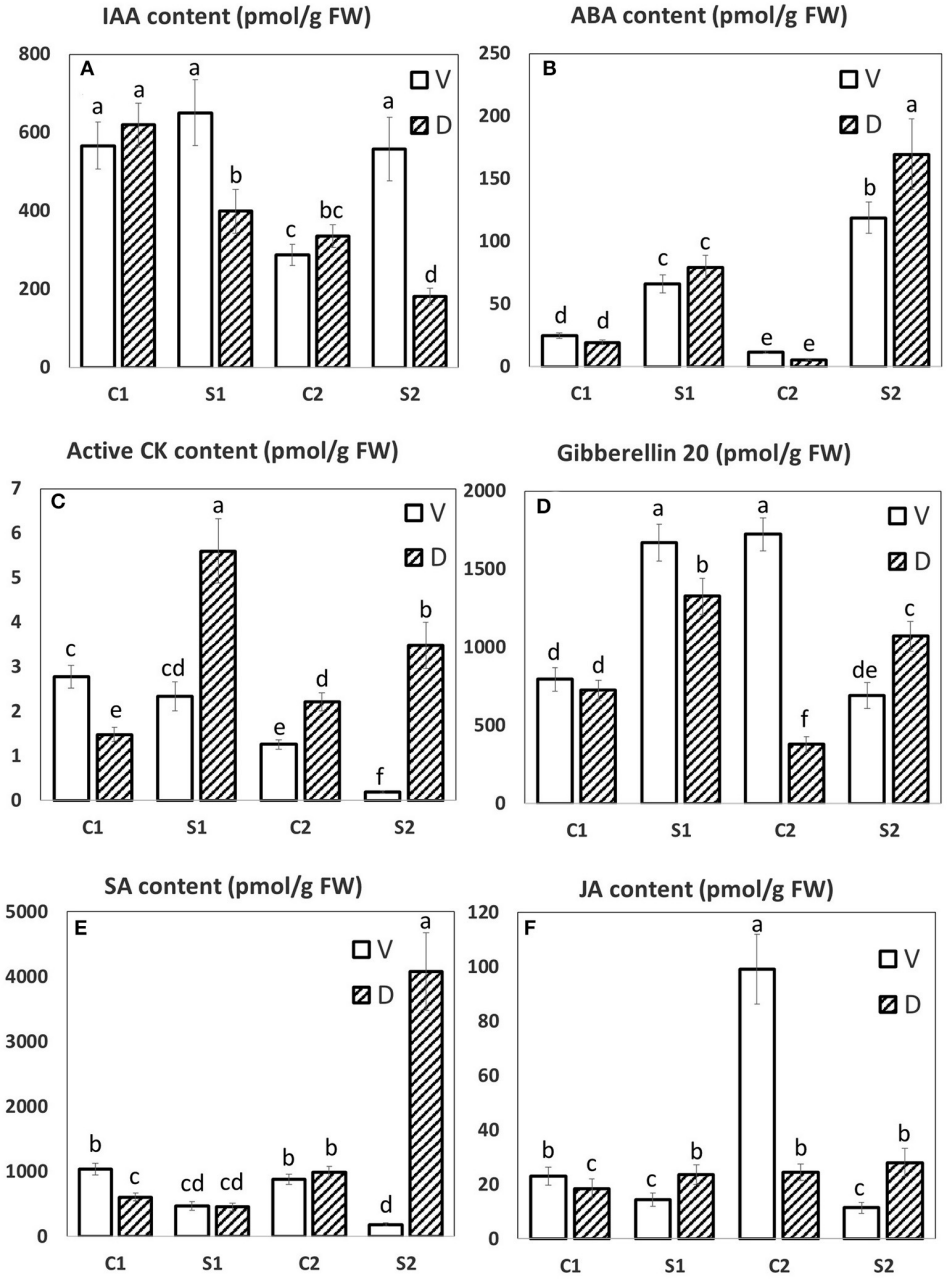
The hormonal profile of MDE. The LC/MS hormonal profile of six selected hormones. **(A)** IAA, indole-3-acetic acid; **(B)** ABA, abscisic acid; **(C)** active CK, active cytokinins; **(D)** Gibberellin 20, Gb_20_; **(E)** SA, salicylic acid; **(F)** JA, jasmonic acid. Means with different letters show significantly different values between treatments at 5% probability. Error bars denote SD.

The phospholipases D alpha 1 (PLD 1; SSP 6705 and 6712; refer to in Figure 7D), phospholipase D alpha 2 (SSP 6716), catalase (SSP 1502; also in Figure 7A), ABA-modulated tyrosine-phosphorylated protein (SSP 4216; also in Figure 7H), both transcription-related proteins (SSP 4711 and 5314), and Clp ATPase (SSP 6710) showed increased protein abundance in DS vs. VS (gene expression of mentioned proteins is shown in Figure 7; mentioned increase in DS is valid for catalase only). Phospholipase D alpha 1 (SSP 6705, 6712) and phospholipase D alpha 2 (SSP 6716; PLD 2) are important enzymes of phospholipid metabolism. Phospholipases D (PLD) and their products, phosphatidic acids, are now considered to be one of the key elements of numerous physiological processes in plants, including the salicylic acid-signaling pathway (Janda et al., 2015). Distefano et al. (2015) showed that pld *Arabidopsis* mutants were more tolerant to severe drought than wild-type plants. This finding suggests that, in wild-type plants, PLD disrupts membranes in severe drought stress and, in the absence of the protein (PLD knockout), might drought-prime the plants, making them more tolerant to severe drought stress. Thus, in contrast to cultivar characteristics and treatment, all PLDs proteins decreased in treated samples, however, D vs. V comparison showed always higher abundance of PLDs in D. These data are in contrast to the PLD 1 gene expression data (Figure 7D) where there is significant increase in all treated samples. However, in V, the SA content (Figure 8E) decreased in treated samples and increased in S2 of D.

### The Comparison Between Leaf Proteome Under Drought and MDE Proteome Under Osmotic Stress

The osmotic stress-affected MDE-derived proteome was compared with the drought-stressed leaf proteome (in the stem-prolongation stage) and analyzed in our previous study (Urban et al., 2017). C vs. D and V were used in both cases. The reason for this comparison has been to reveal possible associations between these two different studies and distinct developmental stages with the aim to evaluate the possible role of MDE in the early selection of more adaptable rapeseed cultivars.

In a simplified way, a comparison of the number of proteins in each functional category between MDE and leaves may reveal a significant increase in protein turnover in MDE. Some protein functional categories, e.g., “protein destination” and “cell structures,” are missing in leaf proteomes. On the other side, ATP interconversion, redox homeostasis, and protein synthesis are higher in the leaf proteome of these two cvs. If we compare individual numbers of increased proteins in each genotype, similarities between MDE and leaf proteome in ATP interconversion and redox homeostasis can be seen in V. Comparison of decreased proteins in individual genotypes for each functional category shows similarities between MDE and leaves proteomes for both cvs., especially in “redox homeostasis” and “stress/defense proteins.”

Some proteins are similar or even identical in both studies (heat map Supplementary Figure 5): glutamine synthetase, lactoylglutathione lyase (glyoxalase), atpA gene product, carbonic anhydrase, malate dehydrogenase 1, oxygen-evolving enhancer protein 1-2, L-ascorbate peroxidase, and glutathione S-transferase. Unfortunately, none of these proteins showed similar patterns in protein accumulation. Interestingly, in MDE, only one small chain RuBisCO (CAA30290.1; SSP 2111; rubisco ssu precursor) was found in contrast to five rbcL (ribulose-1,5-bisphosphate carboxylase/oxygenase large subunit) and nine activases (chloroplast ribulose-1,5-bisphosphate carboxylase/oxygenase activase) in leaves. This can be attributed to the high sugar content (2%) presented in the DM.

Even though drought can be primarily manifested also as osmotic stress (low water potential of soil, increasing xylem sap potential, etc.), a simple relationship between in-pot substrate dry-down and in-sterile cultivation vapor-saturated media is definitely not obvious. Also, at the proteome level, we cannot easily compare these two cultivation methods and developmental stages. Generally, we can conclude that proteome response at the MDE and leaf levels is very different from mean abundances in each category or in comparison of Z-scores. The information about individual protein behavior has already been described in the text above. In this study, the MDE biomass accumulation (higher in D) significantly shows adaptability to osmotic stress at a non-proteomic level. In Urban et al. (2017) study, cultivar D revealed a middle-drought-resistant water-saver strategy in contrast to the drought-susceptible water-spender strategy of cultivar V.

### Comparison/Correlation of Selected Protein Abundance With Relative Gene Expression

The mRNA expression values have provided a lot of information in a broad range of applications, including the diagnosis and the classification of diseases. These results are almost certainly only correlative rather than causative. Most probably, the concentration of proteins and their interactions are the true causative forces in the cells; therefore, the corresponding protein quantities ought to be studied (Greenbaum et al., 2003).

Nine proteins were selected according to their interesting accumulation pattern across genotypes and treatments (Figure 7). The chosen proteins were as follows (SSP, name): 1502, catalase (CAT 4; Figure 7A); 7201, putative lactoylglutathione lyase (L-GUL; Figure 7C); 6705 and 6712, phospholipase D alpha 1 (PLD 1; Figure 7D); 5214, peroxiredoxin antioxidant (PER 1; Figure 7E); 6717, jasmonate inducible protein (JIP; Figure 7F); 4216, ABA-modulated tyrosine-phosphorylated protein (ABA-mtp; Figure 7H); 1608, sulfite reductase (found N.S. in this study; Sred; Figure 7B); 4805, 5-methyltetrahydropteroyltriglutamate-homocysteine methyltransferase (MS 1; Figure 7I); and 4203, glutathione S-transferase (GST; Figure 7G). Expression patterns of individual transcripts are shown in Figure 7 and data in SI 6.

Three of the expression profiles (7DAS) are very similar to protein abundance profiles (7DAS): CAT 4, PER 1, L-GUL (Figures 7A–E).

The other gene expression profiles are not similar to protein accumulation patterns. There is a clear opposite reaction (gene expression vs. protein abundance) visible for Sred, both PLD's, JIP, GST, ABA-mtp, and, partially, also MS 1.

Two of these gene-relative expression profiles (7 DAS) are similar to protein abundance profiles (7 DAS): PER1 and L-GUL. L-GUL acts with Glyoxalase II and detoxification of highly cytotoxic metabolite methylglyoxal accumulated in plant cells due to drought stress (Hasanuzzaman et al., 2017; Askari-Khorasgani and Pessarakli, 2019). Gene expression of L-GUL was upregulated in both cultivars, but D showed a higher level of transcripts (opposite to protein). The accumulation level of L-GUL protein was higher in both treated samples of both cultivars. Protein accumulation of PER1 was accumulated in both cultivars after 7 DAS significantly. This enzyme plays a crucial role in extreme drought stress resistance (Shaikhali et al., 2008; Chen et al., 2016). This corresponds to the gene expression level of PER1. Expression of this gene was upregulated after 7 DAS as well as after 1 DAS. Relative gene expression of this gene in controls was five times higher in the V line in comparison to D (the trend similar in protein accumulation too). Similarly, after 1 DAS, higher levels of PER1 transcript accumulation were found in the V line. After 7 DAS, the level of PER1 transcript accumulation was even increased and was almost the same in both lines. This enzyme is important in the degradation of the reactive oxygen species generated in plants exposed to different types of biotic and abiotic stresses (Tripathi et al., 2009). Generally, lower levels of transcripts in D could indicate a more efficient prevention system of reactive oxygen species generation during early drought stress conditions. Expression of this gene can be thus one of the potential markers for the early selection of more adaptable rapeseed cultivars. Gene expression of PLD 1 increased after 7 DAS as well as after 1 DAS in both lines D and V. In V, accumulation of this protein decreased after 7 DAS (similar decrease observed in VS vs. VC for SSP 6705; SSP 6712 was even missing in VS). This gene is important in the ABA-signaling pathway (Janda et al., 2015; Zhao, 2015). Decreased accumulation of this protein in intolerant V shows that the accumulation efficiency of expressed proteins can be an important part of drought tolerance. The other gene expression profiles are not similar to protein accumulation patterns.

According to Greenbaum et al. (2003), in the case of the lack of obvious correlation between mRNA and protein data, both quantities could be used as independent sources of information for machine-learning algorithms, for example, to predict protein interactions. On this basis, these three genes (L-GUL, PER 1, and PLD 1) can be postulated as suitable for gene-targeting and, also, for early selection of embryos in regard to their osmotic stress adaptability. All genes, PLD 1, PER 1, and L-GUL can be used for early MDE selection as they are stress-related proteins, possibly increasing the adaptability of MDE to osmotic stress (Figures 7C–E). However, CAT 4 and JIP (Figures 7A,F) can be used for characterizing resistant vs. sensitive cultivars.

### Hormonal Analyses Reveal That ABA and Active Cytokinins Play a Crucial Role

The hormonal profile of selected hormones is presented in Figure 8. The complete hormonal profile of PEG-treated MDE upon 1 and 7 DAS can be found in SI 11.

Abscisic acid (ABA) is the most important hormone-controlling plant water loss and, hence, water status and performance in water-limited conditions (Cutler et al., 2010). ABA induces closure of stomata, the crucial water regulation site, as well as stimulates substantial transcription changes, associated with growth suppression and activation of defense. ABA is the key hormone in the responses to abiotic stresses associated with dehydration, not only to drought but also to osmotic stress and cold. The growth on PEG resulted in both MDE tissues, D, and V in early stimulation of ABA content. During the stress progression, ABA levels were further elevated to a significantly higher extent in D line (Figure 8B). ABA elevation was at the early response (after 1 DAS), associated with diminished content of ABA catabolites (DPA, PA, and ABA-GE). During the stress progression, ABA biosynthesis was upregulated to such an extent that catabolite contents increased to maintain ABA at the optimal level.

During abiotic stress responses, ABA exhibits an intensive cross-talk with other plant hormones. For example, jasmonic acid (JA) activates synergistically several branches of the ABA-signaling pathway [especially MYC/MYB and ANAC transcription factors; (de Ollas and Dodd, 2016)]. JA plays an important role, e.g., in the dehydration of desiccation-tolerant plants (Djilianov et al., 2013). Under controlled conditions, D exhibited lower JA content than V (Figure 8F); however, upon PEG treatment, cv. D exhibited significantly higher JA content in comparison to cv. V already after 1 day of stress. After 7-day treatment, D exhibited significant elevation of the active JA conjugate—JA-Ile, while in V content of this compound substantially dropped.

The participation of the other stress hormone—salicylic acid (SA) in drought response may be deduced from the elevation of this hormone during drought stress as well as by the positive effect of exogenous SA application on plant tolerance [(Miura and Tada, 2014); Figure 8E]. Both lines exhibited low SA levels after 1 DAS. During prolonged stress, the D line showed profound SA increase, in contrast to V, which showed SA downregulation. Interestingly, the JA and SA levels in stressed V were almost always lower than control values, opposite to the situation in D.

Also, hormones preferentially associated with stimulation of cell division and growth—auxins and cytokinins (CKs)—were found to participate significantly in stress responses. They affect the growth rate, which is generally diminished upon stress, in order to reallocate the energy sources to defense activation. Regulation of auxin content and transport in osmotic stress response was recently described by Rowe et al. (2016). Auxin (indole-3- acetic acid, IAA, Figure 8A) levels were found to stay low in controls in both cultivars. V cv. maintained relatively high IAA levels during the stress response, while D downregulated IAA content in stress, especially after 7 DAS. The IAA precursor IAN was downregulated in both cultivars at stress conditions. The content of irreversibly inactivated IAA conjugates IAA-Asp correlated with IAA levels (PEG-induced downregulation in D and upregulation in V, respectively). In contrast, the IAA drop was associated with an increase of its deactivation product oxIAA.

CKs affect not only plant growth but also stabilize the photosynthetic system (Boonman et al., 2007; Honig et al., 2018). V diminished significantly the levels of active CKs (trans-zeatin, dihydrozeatin, isopentenyladenine, and cis-zeatin; Figure 8C) during the stress progression, which might indicate “quiescent” stress strategy (diminishing of the energy requirements by growth suppression). A similar strategy was observed, e.g., in plants over-expressing the main CK-degrading enzyme cytokinin oxidase/dehydrogenase (Mackova et al., 2013). In contrast, the D line transiently increased active CK levels, and, even after prolonged stress, it maintained relatively high levels of active CKs, which might have positive effects on the photosynthetic rate and plant metabolism. This result is supported by a high abundance of Rubisco ssu precursor (SSP 2111) and fructose-bisphosphate aldolase 3. This approach has been observed in plants over-expression of CK biosynthetic enzyme isopentenyl transferase under the control of senescence, inducible promoter (Rivero et al., 2007). CK biosynthesis was transiently promoted in both genotypes (after 1 DAS), being diminished after 7 DAS. Downregulation of active CKs in V was accompanied by a stronger increase of CK deactivation products, CK N-glucosides.

Hormonal profiles clearly indicate that D exhibits higher osmotic resistance, which was associated with higher levels of stress hormones ABA, SA, JA, and GA_20_, as well as of active CKs under stress conditions, both from absolute (D vs. V) as from relative (treated vs. control tissues) points of view. In contrast, V quickly downregulates active CK content, which might represent an advantage in case of long-term stress.

## Conclusions

Fertile plants from doubled haploid embryos derived from microspores are both a challenging task and an interesting method to address and understand the effects of abiotic or biotic stress treatments. The likely upcoming new-generation breeding strategies (epigenetic breeding, stress-memory-based breeding, gene editing, associative mapping, etc.) are looking for stable but wide genetic variability within established crops. The MDE seem to be one of the appropriate ways to manage this goal. The technique itself is not easy and far from being used extensively; however, it has already been established for several crops. This proteomic study is among the first steps to demonstrate that MDE is a suitable model for follow-up research aimed at the characterization of new crossings. MDE methodology can likely be used for phenotype-based selection of genotypes resistant to other disrupting effects (other abiotic stresses and their combinations), even though such an effort needs to be confirmed by upcoming studies, including more cultivars or quick selection using biochemical assays.

Cultivar D showed an enhanced biomass accumulation under osmotic stress reflected by the high number of proteins, which belong to energy metabolism (especially glycolysis), redox homeostasis + signaling (phospholipases, MAPK4), transcription, and, also, protein destination, storage, and proteolysis functional categories. Simultaneously, D revealed a unique hormonal profile, high contents of ABA, CKs, SA, JA, and GA_20_ that reflect processes during acclimation of D embryos.

Cultivar D also showed a higher number of unique energy-related proteins and a lower number of down-accumulated proteins compared with V, better ability for protein synthesis, and adjunctive communication between compartments. On the other hand, the V protein profile showed a high need for energy (ATP) and an increased need for nutrients with a significant number of stress-related proteins and cell structure changes. Also, an enhanced number of proteins involved in anaerobic metabolism (e.g., alcohol dehydrogenase) were found in V. In addition, more proteins were generally down-accumulated in sensitive V, which may be connected with the rapid depletion of energy molecules. A similar trend for V was observed also in its leaf proteome upon drought in our previous study Urban et al. (2017).

From a gene expression point of view, we highlighted three genes, which show an interesting pattern of accumulation, and these patterns were in relation to their protein abundances: L-GUL, PLD 1, and PER 1.

Taking these findings together, cv. D showed quick adaptation to osmotically activated PEG-infused cultivation media, while cv. V showed an alert-based response with clear signs of damage. Maintenance of the primary metabolism, oxidative stress response, and signaling seems to be a strategy for D osmotic resistance. On the other hand, susceptibility might be related to the maintenance of the energy-consuming homeostatic equilibrium in V.

## Data Availability Statement

The mass spectrometry proteomics data have been deposited to the ProteomeXchange Consortium via the PRIDE partner repository with the dataset identifier PXD024552.

## Author Contributions

MU: conceptualization, validation, writing of the original draft, reviewing, and editing of the draft. SP: methodology development, protein analysis data, review, and editing of the draft. IH: methodology development, RT-qPCR data, validation of the review, and editing of the draft. RV: methodology development, hormonal analysis, validation, review, and editing of the draft. PD: methodology development and hormonal analysis of data. JR: methodology, review, and editing of the draft. MK: MDE methodology development, validation, supervision, and review of the draft. PV: 2D-DIGE methodology development, validation, supervision, review, and editing of the draft. All authors contributed to the article and approved the submitted version.

## Conflict of Interest

The authors declare that the research was conducted in the absence of any commercial or financial relationships that could be construed as a potential conflict of interest.
