# Supplementary material for: The Resistance of Oilseed Rape Microspore-Derived Embryos to Osmotic Stress Is Associated With the Accumulation of Energy Metabolism Proteins, Redox Homeostasis, Higher Abscisic Acid, and Cytokinin Contents
Source: Front Plant Sci. 2021 Jun 11;12:628167. doi: 10.3389/fpls.2021.628167 (PMC8231708; doi:10.3389/fpls.2021.628167)
Supplement: Supplementary file 1 [file Data_Sheet_1.docx]

Supplementary Material

# Supplementary Data

# Supplementary Figures and Tables

## Supplementary Figures


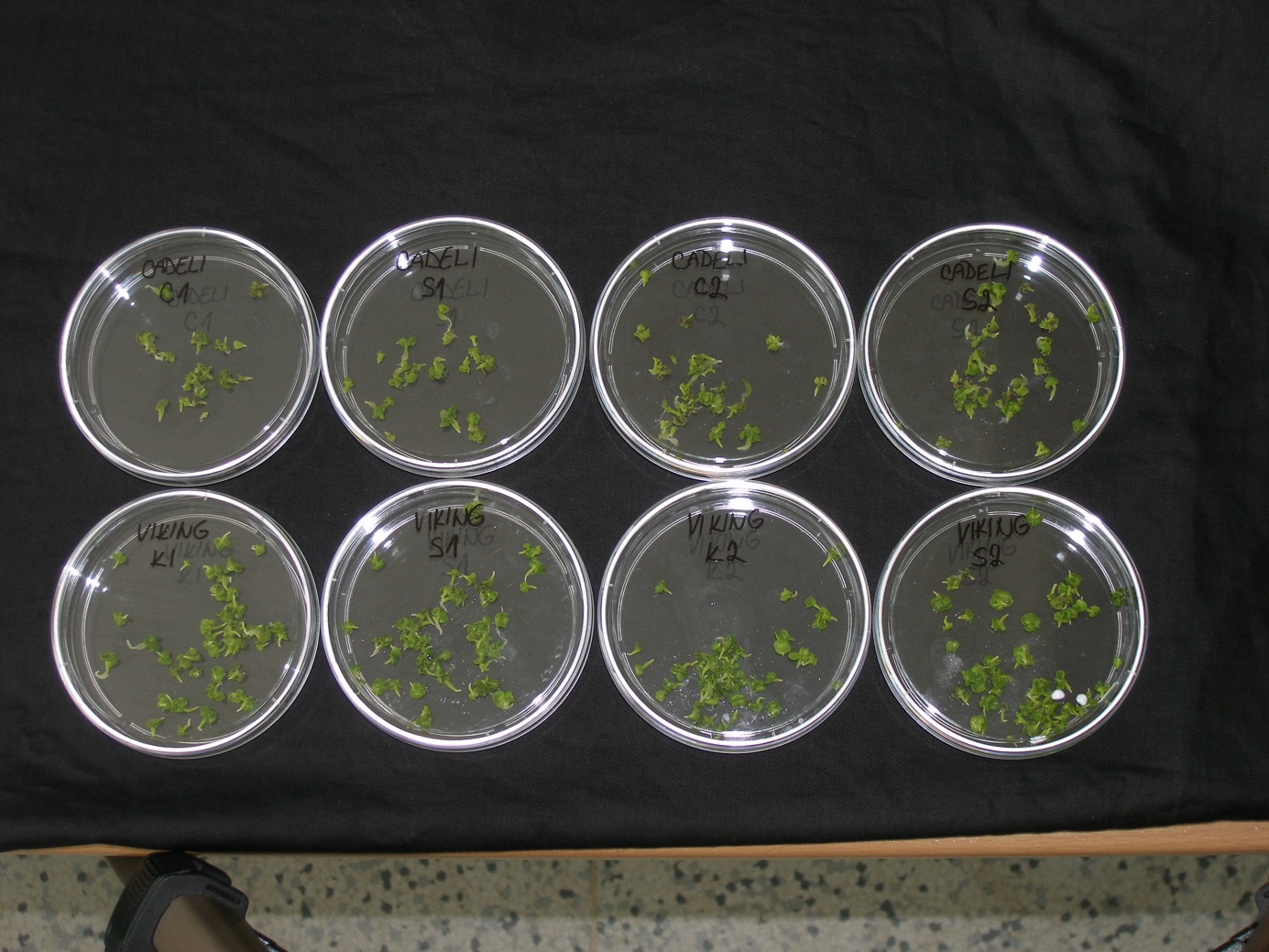


**Suppl. Fig. S-1 Microspore-derived embryos.** Upper row, cv Cadeli; lower row, cv Viking. From left to right as follows: C1, S1, C2, S2. Controls and treated samples (C1 and S1) for the first sampling, respectively (24 hours after stress); C2, S2 – controls and treated samples seven DAS, respectively.


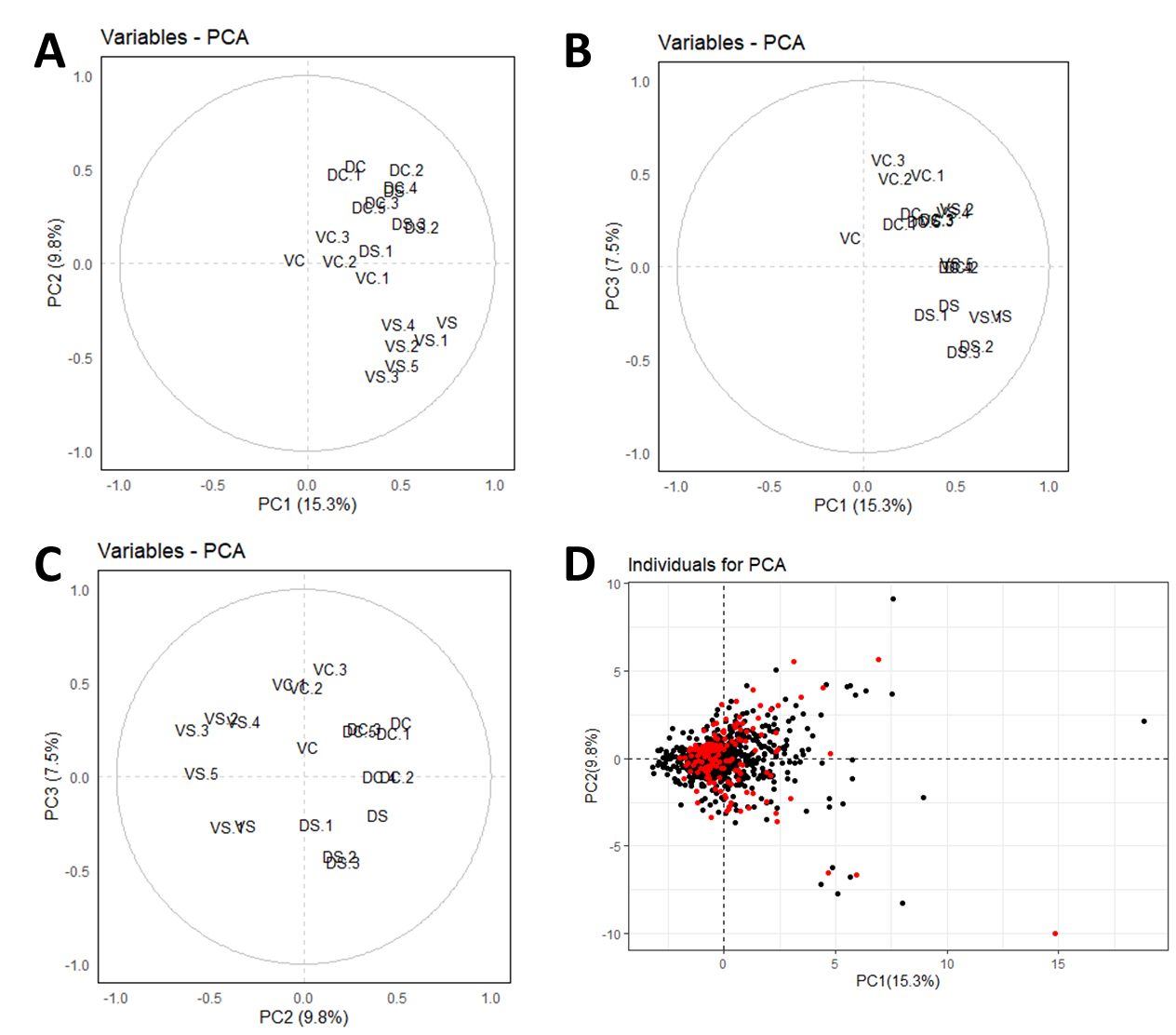


**Suppl. Fig. S-2 PCA of all matched proteins.** Principal component analysis (PCA) of all matched proteins spots (894) chosen for further analysis by PDQuest regarding the individual experimental variants. Analysis was performed using protein abundances between two cvs Cadeli (D) and Viking (V). The projection for variables into factors 1-3, respectively (A, B, and C), the projection of samples (D) into factors. Differentially abundant protein spots revealing high relative abundance reproducibility (less than 50% variation in protein spot relative abundance within the individual replicates in the whole sample set) are indicated in red. DC – Cadeli control, DS – Cadeli treated samples, VC – Viking control, VS – Viking treated samples. To show individual cases the PC1, PC2 and PC3 factors based-projections of data were created and explain almost 33% of variability.


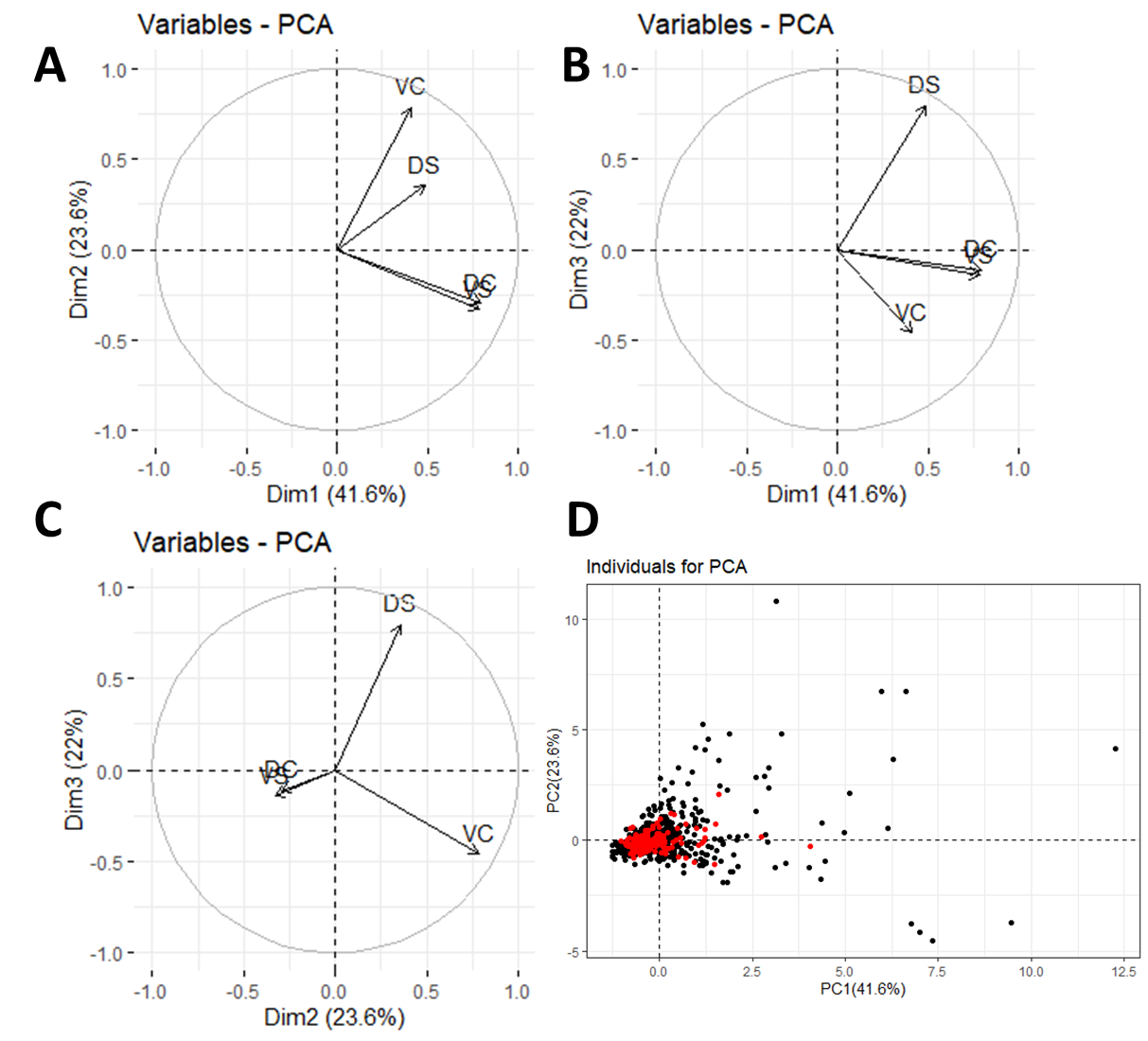


**Suppl. Fig. S-3 PCA of protein averages.** Principal component analysis (PCA) of treatment averages of all normalized proteins (894 spots) between two cvs Cadeli (D) and Viking (V). The projection for variables into factors 1-3, respectively (A, B, and C), the projection of samples (D) into factors. Analysis is based on average protein abundances (see difference to Fig. 3). Differentially abundant protein spots chosen for further analysis by PDQuest (156 spots) revealing high relative abundance reproducibility (less than 50% variation in protein spot relative abundance within the individual replicates in the whole sample set) are indicated in red according to the individual clusters. DC – Cadeli control, DS – Cadeli treated samples, VC – Viking control, VS – Viking treated samples. To show individual cases, the PC1 and PC2 factor based-projections of data were created and explain almost 66% of variability.


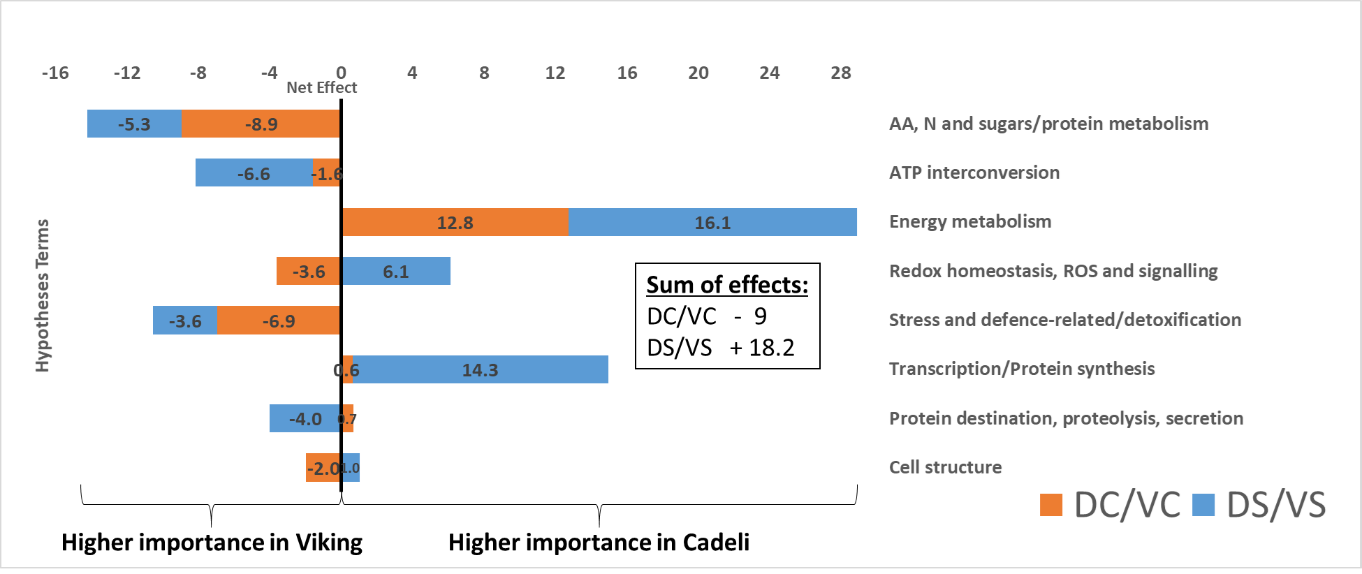


**Suppl. Fig. S-4 GOModeler output.** GOModeler based graphical summary of proteome net effects for each of 8 chosen hypotheses terms (right side) showing control and treated proteins ratios (red, blue groups, respectively). Net effect is a product of quantitative value (logarithm of protein abundance expressed as stress density/control density) with qualitative value (+1, 0, or -1; assessed by GOModeler according to GO annotation of each protein). In the corner, the sum of all net effects for individual combinations is calculated. Positive value (right side of the graph) means higher protein net effect importance in D; negative values means higher net effect importance in V.

#
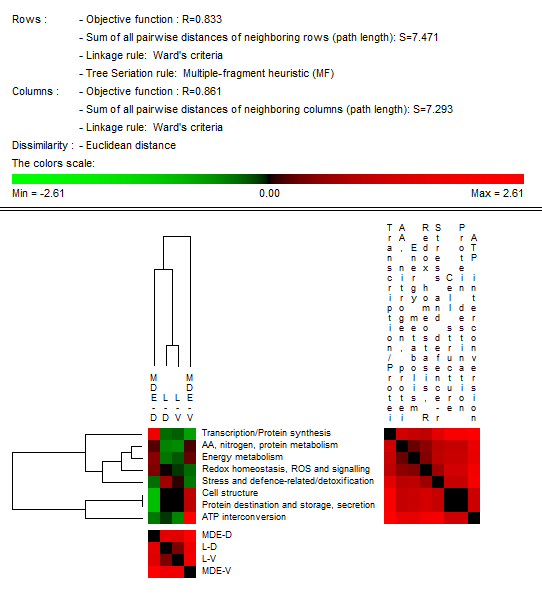


**Suppl. Fig. S-5 Protein abundance vs gene expression heat map.** Heat map (Permut Matrix 1.9.3) comparison of all 8 unique similar proteins between leaf and MDE proteome. MDE D and V (abundance of MDE proteins for Cadeli and Viking, respectively). L D and L V (protein abundance observed in leaf tissues Urban et al. 2017 for Cadeli and Viking, respectively). Proteins are clustered according to the row Z-score values using Euclidean distance and Ward´s minimum criteria, calculated from protein abundances. The red values mean higher protein abundance in the samples, the green values mean lower protein abundance in the sample according to the variant.

## Supplementary Tables

**Tables**

Table S-1. Agronomical characteristics and personal observations of used cultivars

| **Cultivar** | **Country of origin/**  **Company** | **Common characteristics (from official producer websites and GRIN Czech 1.9.1)** | **Our personal observations upon cultivars** |
| --- | --- | --- | --- |
| **Viking (V; line)** | Germany  NPZ (Norddeutsche Pflanzenzucht)  (year of registration 2002) | <https://grinczech.vurv.cz/gringlobal/accessiondetail.aspx?id=60847>  - early cultivar (flowering time)  - small/intermediate size (low plant, smaller leaves)  - very rapid autumn development and rosette formation  - quick spring regeneration  - very high tolerance to lodging before harvest  - low/lower cold and frost resistance  - strong root system and rapid root development  - resistance to fungal disease and pests  - middle seed yield and low TSW  - glucosinolates content lowest within known rapeseed cultivars and very low erucic acid content  - high oil content in seeds  - more suitable for warm agriculture areas | Higher germination rate in high temperature (42°C day, 20°C night). Highest osmotic adjustment of leaves in drought stress. LOW root:shoot ratio under drought stress. Relative expression of COR25, ERD10 and ERD15 dehydrins higher in controls than in drought stressed plants. |
| **Cadeli**  **(D; line)** | France  Monsanto SAS  Monsanto Technology LLC  (year of registration 2007) | <https://grinczech.vurv.cz/gringlobal/accessiondetail.aspx?id=60875>  - intermediate/late cultivar (flowering time)  - middle size (middle plant, middle leaves)  - rapid autumn development and rosette formation  - long flowering period  - middle resistance to lodging before harvest  - low/lower cold and frost resistance  - strong root system and rapid root development  - good/very good resistance to fungal disease and pests  - high oil content (very high oleic acid = oil stability)  - higher adaptability to drought in warmer conditions  - low seed yield and high TSW  - very low glucosinolates content  - more suitable for warm and drier agriculture areas | Higher germination rate in high temperature (42°C day, 20°C night). High root:shoot ratio under drought stress. Middle dehydrins content at low (4°C) temp. Relative expression of COR25, and ERD10 dehydrins higher in controls than in drought stressed plants comparable to Viking. |

Table S-2. Clustering of proteins according to their abundance between variants

| **Cluster number** | **Color of cluster** | **Uniquely accumulated proteins in variants** | **Number of spots** | **Most abundant protein processes – protein functional categories (DIFFP)** |
| --- | --- | --- | --- | --- |
| **1** | light green | DC | 3 | 3 (2x),5 |
| **2** | light yellow | DS | 6 | 1,2,3(2x),4,6 |
| **3** | Red | DC+DS | 12 | 1(2x),3(7x),5,6,7 |
|  |  | cv Cadeli | 21 | 1(3x),2,3(11x),4,5(2x),6,7 |
| **4** | dark green | VC | 7 | 1(2x),3(2x),4,5,8 |
| **5** | dark yellow | VS | 5 | 1,2,3(2x),7 |
| **6** | Blue | VC+VS | 7 | 1(3x),3(3x),5 |
|  |  | cv Viking | 19 | 1(6x), 2, 3(7x), 4,5(2x), 7,8 |
| **7** | Black | DC+VC | 3 | 1,3,5 |
| **8** | Grey | DS+VS | 5 | 3(2x),4(3x) |
| **9** | White | miscellaneous | 10 | 1(2x),3,4(3x),5(3x),7 |

Table S-3. The list of primer sequences used for qRT-PCR

| **Target gene** | **Primer (forward)** | **Amplicon size** | **NCBI accession number** |
| --- | --- | --- | --- |
| **Brassica rapa putative lactoylglutathione lyase** | F: ACAAAGGGCAACGCATATGC | 62 bp | XM_009112446.3 |
|  | R: TTCAGCGCTTTTGTACACATCA |  |  |
| **Brassica napus catalase-2-like** | F: CGCTCTCAAACCAAACCCAA | 83 bp | XM_022706149.1 |
|  | R: TTAAGCTCTCAGGGTGGTGG |  |  |
| **Brassica napus assimilatory sulfite reductase** | F: TGAGCTTGGTCTAGTGGGTG | 75 bp | XM_022693648.1 |
|  | R: ATCTGTGTCTGGTTCGGTGT |  |  |
| **Brassica rapa glutathione S-transferase U5-like** | F: CCGAGCAGACAAGAGAACTG | 109 bp | XM_022720190.1 |
|  | R: ATCACACTTCCGGCGACTAA |  |  |
| **Brassica napus uncharacterized protein At5g02240-like** | F: AAAGAAGGAGGTGCACGAGA | 120 bp | XM_013812817.2 |
|  | R: CTCCAACAGCAATGCCTGAA |  |  |
| **Brassica napus 5-methyltetrahydropteroyltriglutamate--homocysteine methyltransferase 1** | F: CGCCCAGAAGATCGTTGAAG | 122 bp | XM_022709081.1 |
|  | R: TGGTGACTCTTGGGGAAGAC |  |  |
| **Brassica napus 1-Cys peroxiredoxin PER1** | | 73 bp | NM_001315889.1 |
| **Brassica napus L-ascorbate peroxidase 1, cytosolic-like** | F: TGTGACCACTTGAGGCAG | 104 bp | XM_013785685.2 |
|  | R: ATCCTTGTGGCATCTTCCCA |  |  |
| **B. napus mRNA for jasmonate inducible protein** | F: CTGGAGCTGTATGGGACGAT | 70 bp | Y11483.1 |
|  | R: CCATCTGTGCCTTGTCCAAC |  |  |
| **Brassica napus phospholipase D1** | F: CATGTTCACGCACCATCAGAAG | 102 bp | XM_013841229.2 |
|  | R: GAGTTTTGTTGGTGGGATCGA |  |  |
